# Fruit flies exploit behavioral fever as a defense strategy against parasitic insects

**DOI:** 10.1126/sciadv.adw0191

**Published:** 2025-06-11

**Authors:** Yifeng Sheng, Zixuan Xu, Yang Li, Jiani Chen, Lan Pang, Yueqi Lu, Zhi Dong, Qichao Zhang, Junwei Zhang, Ting Feng, Wenqi Shi, Ying Wang, Xuexin Chen, Xing-Xing Shen, Jianhua Huang

**Affiliations:** ^1^ Zhejiang Key Laboratory of Biology and Ecological Regulation of Crop Pathogens and Insects, Institute of Insect Sciences, College of Agriculture and Biotechnology, Zhejiang University, Hangzhou 310058, China.; ^2^ Ministry of Agriculture Key Laboratory of Molecular Biology of Crop Pathogens and Insects, Zhejiang University, Hangzhou 310058, China.; ^3^ Centre for Evolutionary & Organismal Biology, Zhejiang University, Hangzhou 310058, China.

## Abstract

Behavioral fever, a thermoregulatory response in which ectothermic animals seek warmer environments to elevate body temperature and combat parasite infections, is well documented against microparasites. However, its role and mechanisms against macroparasites remain largely unknown. Here, we show that *Drosophila* hosts use behavioral fever to defend against *Leptopilina* parasitoid wasps. This thermal preference increases wasp mortality and enhances host survival. We find that behavioral fever is mediated by up-regulation of *Heat shock protein 70* (*Hsp70*) genes in infected hosts as *Hsp70* loss abolishes behavioral fever, whereas its overexpression induces heat-seeking behavior. We further find that behavioral fever up-regulates immune genes in infected hosts, including 12 *antimicrobial peptide* (*AMP*) genes, which disrupt the gut microbiota homeostasis of parasitoid wasps and, in turn, lead to substantial wasp mortality. Our findings elucidate the detailed mechanisms of behavioral fever in *Drosophila* hosts, advancing our understanding of ectothermic animal defenses against macroparasites.

## Teaser

Fruit flies use behavioral fever to combat parasitoid wasps and boost survival.

## INTRODUCTION

In nature, animals are commonly infected by parasites, including microparasites (e.g., viruses, bacteria, fungi, and protozoa) and macroparasites (e.g., parasitic helminths, parasitic ticks, and parasitic insects) (*1*–*3*). Almost all parasites can exploit the host and, in doing so, cause harm to or even the death of host animals. In response, host animals have developed effective strategies to fight parasitic infections. Fever is an evolutionarily conserved immune response of infected animals, including endotherms (e.g., mammals and birds) and ectotherms (e.g., fishes, amphibians, reptiles, and insects). Unlike endothermic animals, which exhibit a metabolic increase in their body temperature (*4*–*5*), ectothermic animals lack such intrinsic thermogenesis processes and therefore manipulate their body temperature by moving to warmer places (*6*–*12*). This fascinating strategy, known as behavioral fever, is widely reported in ectothermic animals when they are infected by microparasites. For example, the desert iguana (*Dipsosaurus dorsalis*), the bluegill sunfish (*Lepomis macrochirus*), and the largemouth black bass (*Micropterus salmoides*) have been shown to exhibit a behavioral fever when infected by a Gram-negative bacterium, *Aeromonas hydrophila* (*6*–*9*); the common carp (*Cyprinus carpio*) exhibits behavioral fever in response to cyprinid herpesvirus infection (*11*); and the locust *Locusta migratoria* has the capacity to develop a behavioral fever to reduce *Metarhizium anisopliae* fungal infection (*12*). However, the lack of empirical studies has limited our understanding of whether ectothermic animals exhibit behavioral fever when they are infected by macroparasites. Moreover, the mechanisms underlying behavioral fever in ectothermic animals remain poorly understood.

Parasitoid wasps represent one of the largest groups of macroparasites, with estimates of >150,000 species accounting for 20% of the insect species on earth (*13*). Parasitoid wasp-host interactions can constitute an arms race: Although the parasitoid wasp tends to improve its parasitic success, the host is permanently under selection for increased resistance. As such, parasitoid wasps have evolved multiple effectors to modulate the host’s immunity and nutritional metabolism to create favorable conditions for successful parasitism. The effectors include venoms, polydnaviruses, teratocytes, and larval secretions (*14*–*16*). On the other hand, host animals constantly strengthen their immune systems and recruit several foreign genes via horizontal gene transfer to protect against parasitoid wasp infection (*17*, *18*). Recently, *Drosophila melanogaster* hosts were found to undergo intriguing behavioral changes to defend against parasitoid wasp infection, including altered food preferences, reduced oviposition rates, and accelerated mating behavior (*19*–*22*). For example, one study has shown that infected *D. melanogaster* larvae preferentially consume toxic levels of alcohol-rich food because the benefit of alcohol-mediated wasp death outweighs the cost to the hosts of alcohol consumption, which is an example of therapeutic self-medication (*22*). Therefore, the *Drosophila* and parasitoid wasp system provides a valuable model for studying the behavioral immune response of ectothermic animals in response to macroparasite infection.

Here, we designed behavioral assays to detect the thermal preferences of *D. melanogaster* hosts when they were infected by four *Leptopilina* parasitoid wasp species (*L. heterotoma*, *L. boulardi*, *L. myrica*, and *L. syphax*). Thus, we aimed to answer three questions: (i) Do ectothermic animals exhibit behavioral fever as a defense strategy against infection by macroparasites (e.g., parasitoid wasps)? (ii) What factors trigger behavioral fever in infected animals? (iii) How does behavioral fever mount a successful immune response and benefit host survival?

## RESULTS

### Fruit fly hosts exhibit behavioral fever in response to parasitoid wasp infection

**Fig. 1.**
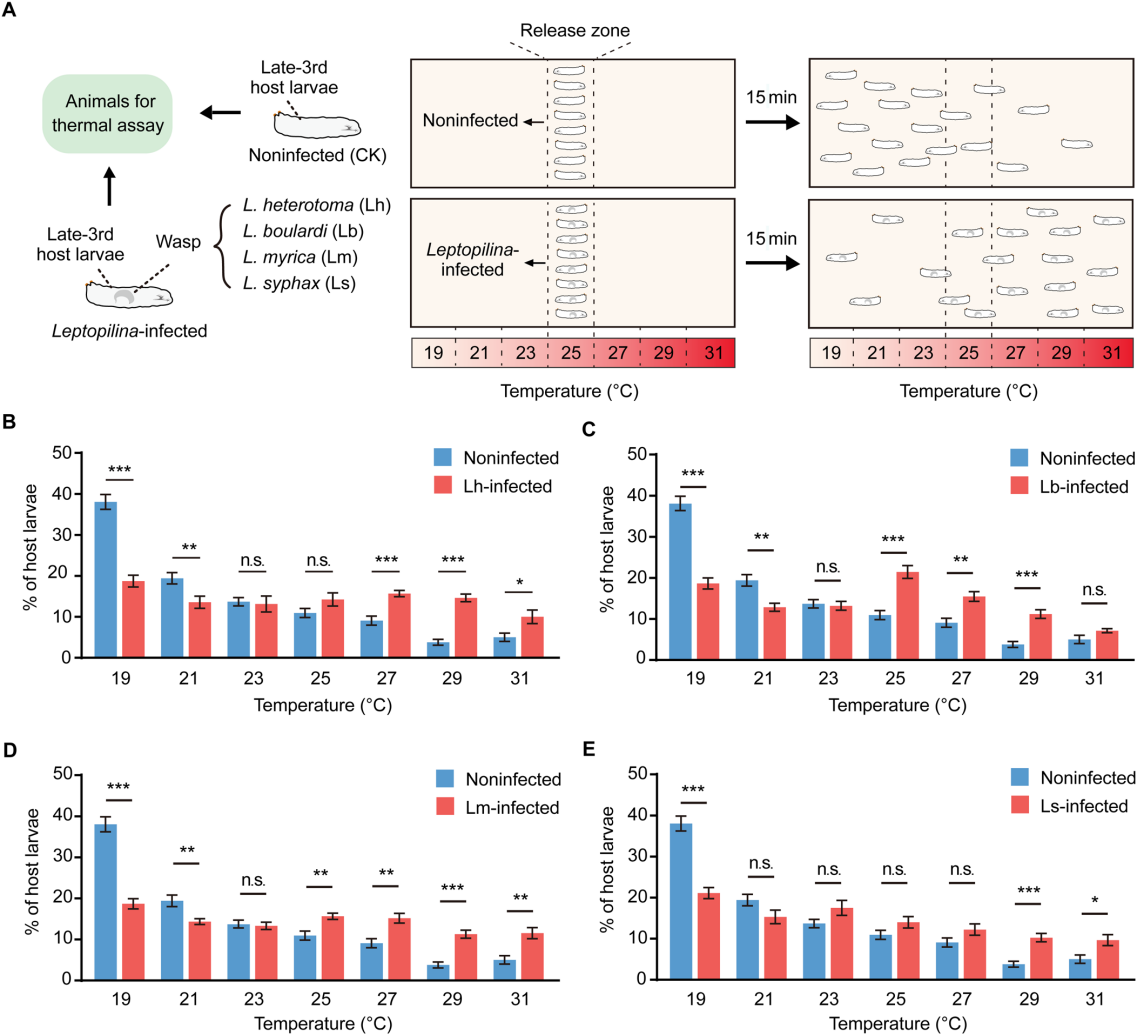
Thermal preferences of *Leptopilina*-infected host larvae. (**A**) Schematic diagram of temperature gradient assays for *D. melanogaster* host larvae. (**B** to **E**) Thermal preference of late-third instar *D. melanogaster* host larvae infected by *L. heterotoma* (Lh-infected) (B), *L. boulardi* (Lb-infected) (C), *L. myrica* (Lm-infected) (D), or *L. syphax* (Ls-infected) (E). Noninfected late-third instar host larvae (noninfected) were used as controls. Experiments were performed with eight biological replicates. The data are presented as the means ± SEMs. Significance was determined by two-tailed unpaired Student’s *t* test (*P < 0.05; **P < 0.01; ***P < 0.001; n.s., not significant).

We designed an apparatus that allows the animals to choose their preferred temperature within a continuous linear gradient (19° to 31°C range) to investigate whether *D. melanogaster* hosts change their thermal preference when they are infected by parasitoid wasps (fig. S1, A and B). On the basis of the parasitic performance on *D. melanogaster* hosts, three different *Leptopilina* wasp species (*L. heterotoma*, *L. boulardi*, and *L. myrica*) were allowed to infect second instar *D. melanogaster* host larvae [60 hours after egg laying (AEL)], and *L. syphax* was allowed to infect first instar *D. melanogaster* host larvae (30 hours AEL). The generated early-third instar larvae (72 hours AEL) and late-third instar larvae (120 hours AEL) were used to conduct the thermal preference experiments (Fig. 1A and fig. S2). Consistent with previous observations (*23*), the noninfected early-third instar *D. melanogaster* host larvae preferred the 25°C region, whereas the noninfected late-third instar *D. melanogaster* host larvae preferred the 19°C region of the gradient. Compared to the noninfected controls, the infected early-third instar host larvae presented no distinguishable thermal preference changes (fig. S2); however, the infected late-third instar host larvae presented increased accumulation in the hot regions (25° to 31°C) (Fig. 1, B to E). Specifically, the late-third instar host larvae infected by *L. heterotoma* prefer the high-temperature regions of 27°, 29°, and 31°C (Fig. 1B); the late-third instar host larvae infected by *L. boulardi* prefer the high-temperature regions of 25°, 27°, and 29°C (Fig. 1C); the late-third instar host larvae infected by *L. myrica* prefer the high-temperature regions of 25°, 27°, 29°, and 31°C (Fig. 1D); and the late-third instar host larvae infected by *L. syphax* prefer the high-temperature regions of 29° and 31°C (Fig. 1E). These results showed that *D. melanogaster* hosts maintained their thermal preference during the early infection stage but altered it during the later infection stage.

We designed another two-way choice assay in which the 19°C region was selected over the 29°C region to confirm whether the preferred temperature of late-third instar *D. melanogaster* host larvae changes due to parasitoid wasp infection (figs. S1C and S3A). Consistent with the linear gradient assays, we found that the infected *D. melanogaster* host larvae exhibited behavioral fever instead of the normally preferred 19°C region (fig. S3B). Specifically, 55.51 ± 1.10% of *L. heterotoma*–infected host larvae, 44.76 ± 2.31% of *L. boulardi*–infected host larvae, 45.55 ± 2.32% of *L. myrica*–infected host larvae, and 40.50 ± 1.22% of *L. syphax*–infected host larvae displayed a preference for the 29°C region compared to the noninfected host larvae, with only 14.33 ± 1.13% choosing the 29°C region (fig. S3B). In addition, we confirmed that mechanical stress from fine needle pricking, which mimics the ovipositor puncture of parasitoid wasps, did not alter the thermal preference of late-third instar *D. melanogaster* host larvae (fig. S4).

Collectively, these results suggest that the observed preference for higher temperatures in infected *D. melanogaster* hosts likely represents a behavioral fever defense strategy against parasitoid wasp infection. Furthermore, the fact that *D. melanogaster* hosts exhibit behavioral fever specifically during the later infection stage may reflect an adaptive strategy to minimize fitness costs as prolonged exposure to high temperatures could have detrimental effects on host survival and development.

### *Hsp70* gene expression is elevated in infected fruit fly hosts

We tested the expression profiles of thermal preference regulatory genes, including *painless*, *transient receptor potential cation channel A1* (*trpA1*), *neither inactivation nor afterpotential E* (*ninaE*), *Rhodopsin 5* (*Rh5*), *Rhodopsin 6* (*Rh6*), *Ionotropic Receptor 21a* (*IR21a*), *Ionotropic Receptor 25a* (*IR25a*), *brivido-1* (*brv1*), *brivido-2* (*brv2*), *brivido-3* (*brv3*), *inactive* (*iav*), and *Gustatory Receptor 28b* (*GR28b*) (*23*–*30*), to determine the key factors that manipulate the thermal preference of infected fly hosts. The quantitative real-time polymerase chain reaction (PCR) (qPCR) analysis showed no significant changes in the expression of these genes between the infected host larvae and the noninfected host larvae (fig. S5), indicating that none of the renowned regulatory genes related to thermal preference are involved in the behavioral fever response.

**Fig. 2.**
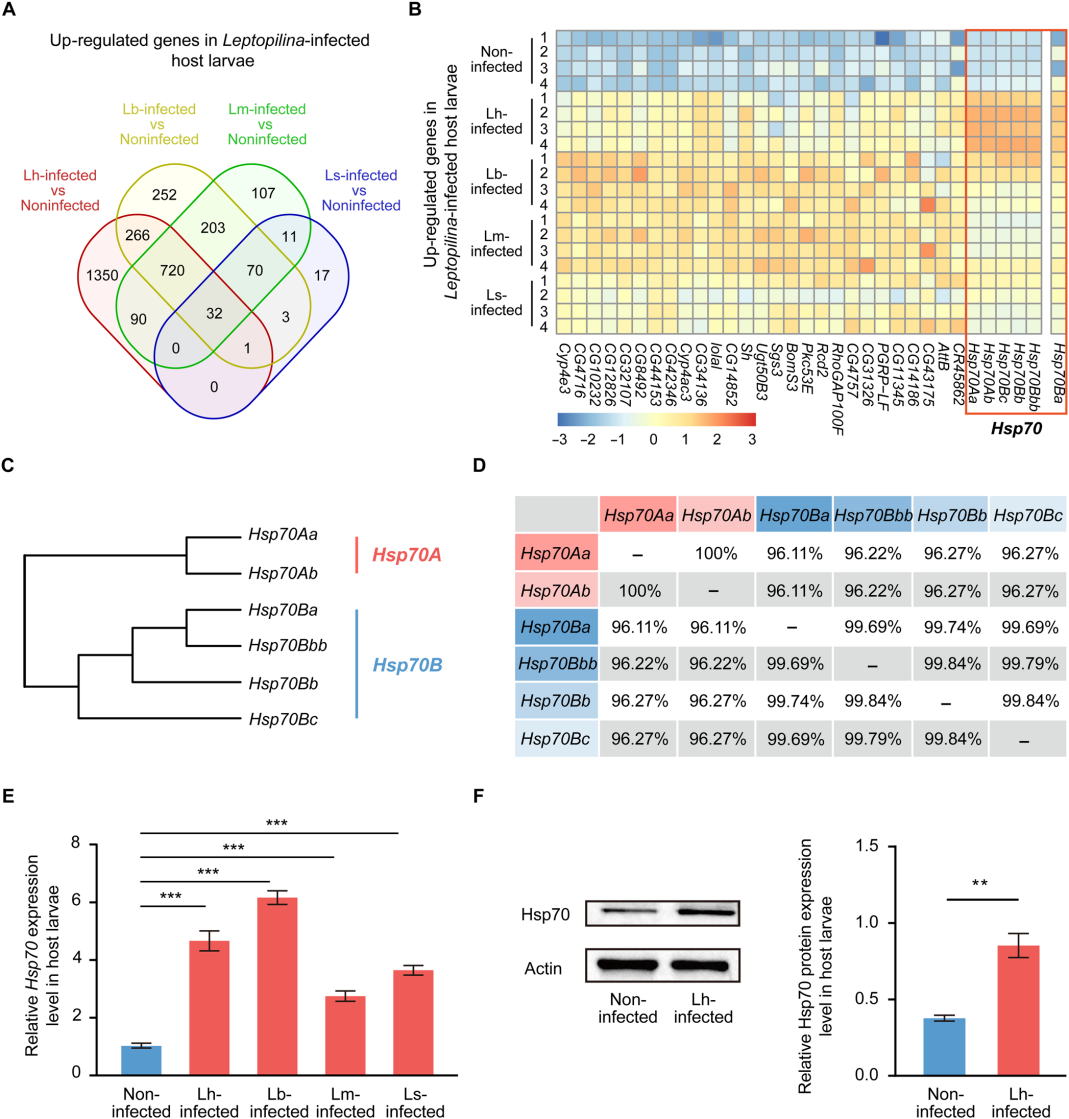
Increased expression of Hsp70 in infected fly hosts. (**A**) Venn diagram illustrating the up-regulated DEGs identified via a transcriptomic analysis of *Leptopilina*-infected late-third instar host larvae compared with noninfected late-third instar host larvae. Each circle represents a comparison, with the overlaps indicating common up-regulated DEGs among different comparisons. Noninfected, noninfected host larvae; Lh-infected, *L. heterotoma*-infected host larvae; Lb-infected, *L. boulardi*–infected host larvae; Lm-infected, *L. myrica*–infected host larvae; Ls-infected, *L. syphax*–infected host larvae. (**B**) Expression profiles of up-regulated DEGs in host larvae infected with the four *Leptopilina* wasps. Six *Hsp70* genes are highlighted in an orange box. The red and blue colors represent high to low expression levels, respectively, based on the FPKM values. (**C**) Phylogenetic tree of the six *Hsp70* genes in *D. melanogaster* hosts. The *Hsp70A* subgroup includes the *Hsp70Aa* and *Hsp70Ab* genes; the *Hsp70B* subgroup includes the *Hsp70Ba*, *Hsp70Bbb*, *Hsp70Bb*, and *Hsp70Bc* genes. (**D**) Comparison of the coding sequence identities among the six *Hsp70* genes. (**E**) Relative mRNA levels of *Hsp70* genes in *Leptopilina*-infected hosts. Noninfected hosts were used as controls. The experiments were performed with three biological replicates. The data are presented as the means ± SEMs. Significance was determined by one-way ANOVA with Tukey’s multiple comparison test (***P < 0.001). (**F**) Immunoblot analysis of Hsp70 and Actin (loading control) levels in noninfected and Lh-infected host larvae. Hsp70 protein levels were normalized to the loading control to compare changes in relative expression. The experiments were performed with three biological replicates. The data are presented as the means ± SEMs. Significance was determined by two-tailed unpaired Student’s *t* test (**P < 0.01).

Thus, we generated transcriptome data from noninfected *D. melanogaster* larvae and *D. melanogaster* larvae infected with *L. heterotoma*, *L. boulardi*, *L. myrica*, or *L. syphax* parasitoid wasps. We found that 32 genes were significantly up-regulated and 23 genes were significantly down-regulated in all four parasitoid wasp-infected hosts compared with noninfected hosts (Fig. 2A, fig. S6, and tables S1 and S2). Notably, a group of *Heat shock protein 70* (*Hsp70*) genes attracted our attention because they accounted for 5 of the 32 up-regulated genes, including *Hsp70Aa*, *Hsp70Ab*, *Hsp70Bbb*, *Hsp70Bb*, and *Hsp70Bc* (Fig. 2B). The *Hsp70* gene family is evolutionarily conserved and generally is present in multiple copies in different animals. In *D. melanogaster*, a total of six *Hsp70* copies from the *Hsp70A* and *Hsp70B* subgroups were identified (Fig. 2C). Specifically, two genes of the *Hsp70A* subgroup (*Hsp70Aa* and *Hsp70Ab*) are located at cytological locus 87A on the right arm of the third chromosome and share exactly the same coding sequence; four genes of the *Hsp70B* subgroup (*Hsp70Ba*, *Hsp70Bbb*, *Hsp70Bb*, and *Hsp70Bc*) are located at cytological locus 87C on the right arm of the third chromosome and share more than 99.69% identity of the coding sequences (Fig. 2D and figs. S7 and S8A) (*31*). Similar to the elevated expression of *Hsp70Aa*, *Hsp70Ab*, *Hsp70Bbb*, *Hsp70Bb*, and *Hsp70Bc* in infected fruit fly hosts, the expression of the remaining gene, *Hsp70Ba*, was also significantly increased in *L. heterotoma*–infected host larvae (fold change = 51.27, *P* < 0.001) and *L. boulardi*–infected host larvae (fold change = 9.19, *P* < 0.05) and increased in *L. myrica*–infected host larvae (fold change = 3.63, *P* = 0.078) and *L. syphax*–infected host larvae (fold change = 3.41, *P* = 0.067), although these changes were not significant (Fig. 2B and table S1).

We next used a pair of conserved primers to verify the expression of *Hsp70* genes in *Leptopilina*-infected hosts through qPCR (fig. S7). Similar to the results of the transcriptomic analysis, the qPCR results confirmed that the expression of *Hsp70* genes was significantly increased in fruit fly hosts after parasitoid wasp infection (Fig. 2E). Furthermore, the results of immunoblotting assays revealed that the Hsp70 protein level was also higher in infected fruit fly hosts than in noninfected hosts (Fig. 2F).

Collectively, these results indicate that Hsp70 expression is highly elevated in parasitoid wasp-infected hosts, suggesting an unusual role of Hsp70 induction in infected host animals.

### The induction of *Hsp70* expression is necessary for host behavioral fever

**Fig. 3.**
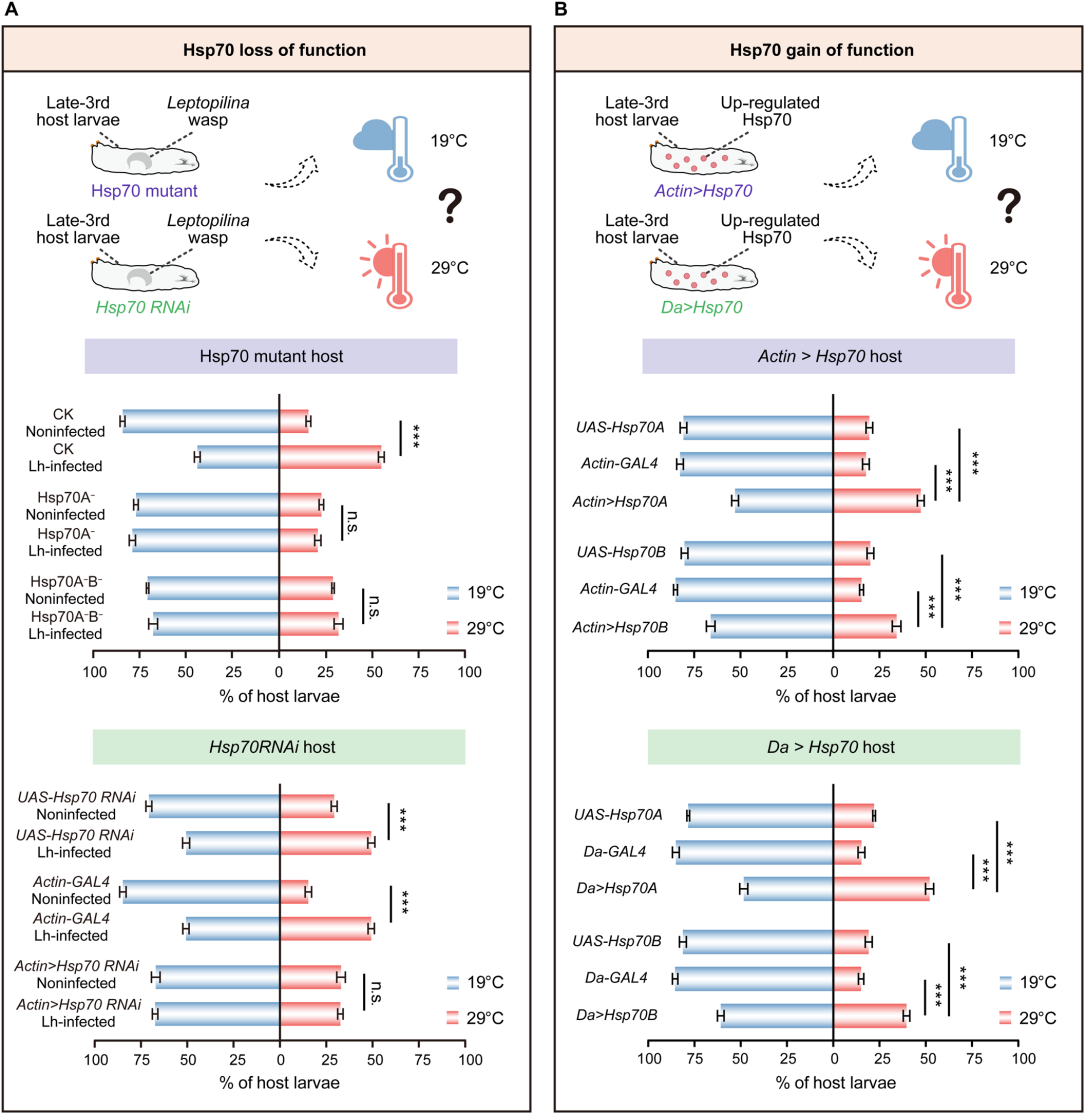
Hsp70 induction is important to trigger the behavioral fever of infected fly hosts. (**A**) Thermal preference (19° versus 29°C) of *D. melanogaster* host larvae with Hsp70 loss-of-function genotypes. Lh-infected, *L. heterotoma*–infected late-third instar host larvae; Noninfected, noninfected late-third instar host larvae. The host genotypes are CK (*w^1118^*), *Hsp70A^−^* (mutation of the *Hsp70A* subgroup), *Hsp70A^−^B^−^* (mutation of all *Hsp70* genes), *UAS-Hsp70 RNAi*, *Actin-GAL4*, and *Actin>Hsp70 RNAi*. Experiments were performed with at least 10 biological replicates. The data are presented as the means ± SEMs. Significance was determined by two-tailed unpaired Student’s *t* test (***P < 0.001; n.s., not significant). (**B**) Thermal preference (19° versus 29°C) of *D. melanogaster* host late-third instar larvae with Hsp70 gain-of-function genotypes. The host genotypes are *UAS-Hsp70A*, *Actin-GAL4*, *Actin>Hsp70A*, *UAS-Hsp70B*, *Actin>Hsp70B*, *Da-GAL4*, *Da>Hsp70A*, and *Da>Hsp70B*. Experiments were performed with at least 10 biological replicates. The data are presented as the means ± SEMs. Significance was determined by one-way ANOVA with Tukey’s multiple comparison test (***P < 0.001).

We then hypothesized that Hsp70 induction was required for infected hosts to choose a hot temperature. We tested the performance of *L. heterotoma*–infected *D. melanogaster* larvae in two-way thermal preference assays in which Hsp70 function was eliminated to assess the potential importance of Hsp70 in thermotactic behavior (Fig. 3A). Two mutant alleles, Hsp70A^−^ and Hsp70A^−^B^−^, were used to eliminate Hsp70 function (*31*). Hsp70A^−^ deletes the two genes of the *Hsp70A* subgroup, and Hsp70A^−^B^−^ deletes all six *Hsp70* genes (fig. S8B). Similar to the noninfected control hosts, the two Hsp70 mutant larvae pursued a lower temperature and congregated in the 19°C region (Fig. 3A). Notably, *L. heterotoma*–infected Hsp70A^−^ and *L. heterotoma*–infected Hsp70A^−^B^−^ mutant larvae nearly lost their ability to undergo behavioral fever compared with the infected control hosts, which congregated at the 29°C region (Fig. 3A). We also performed RNA interference (RNAi)–mediated knockdown of *Hsp70* genes in *L. heterotoma*–infected host larvae using a ubiquitous *GAL4* line (*Actin-GAL4*) (fig. S8C). As expected, the knockdown of *Hsp70* genes resulted in a significant reduction in the proportion of *L. heterotoma*–infected host larvae that preferred the 29°C region compared with the *L. heterotoma*–infected genetic controls (*Actin-GAL4* and *UAS-Hsp70 RNAi*) (Fig. 3A). These results suggest that Hsp70 is important for parasitoid wasp-infected fruit fly hosts to change their preferred temperature of 19°C to a much higher temperature of 29°C.

We determined whether ectopic expression of *Hsp70* genes is sufficient to trigger behavioral fever by expressing *Hsp70A* (A isoform, in which *Hsp70Aa* and *Hsp70Ab* have 100% amino acid identity) and *Hsp70B* (B isoform, in which *Hsp70Ba*, *Hsp70Bbb*, *Hsp70Bb*, and *Hsp70Bc* have at least 99.53% amino acid identity) using two ubiquitous *GAL4* lines (*Actin-GAL4* and *Da-GAL4*), respectively (Fig. 3B and fig. S9, A and B). The results of qPCR with a pair of conserved primers revealed that the expression of *Hsp70* genes was significantly up-regulated in *Hsp70A-* and *Hsp70B*-overexpressing hosts (*Actin>Hsp70A*, *Da>Hsp70A*, *Actin>Hsp70B*, and *Da>Hsp70B*) (fig. S9C). We next found that *D. melanogaster* host larvae overexpressing *Hsp70A* or *Hsp70B* presented a significantly increased preference for the high temperature (29°C) (Fig. 3B). Specifically, 47.16 ± 1.84% of *Actin>Hsp70A* and 51.78 ± 2.20% of *Da>Hsp70A* larvae in the *Hsp70A*-overexpressing groups preferred the 29°C region compared with the control groups (*UAS-Hsp70A*: 19.40 ± 1.79%, *Actin-GAL4*: 17.52 ± 1.81%; *UAS-Hsp70A*: 21.86 ± 0.75%, and *Da-GAL4*: 14.91 ± 1.85%); meanwhile, 34.20 ± 2.34% of *Actin>Hsp70B* and 39.41 ± 1.79% of *Da>Hsp70B* larvae in the *Hsp70B*-overexpressing groups preferred the 29°C region compared with the control groups (*UAS-Hsp70B*: 19.76 ± 1.80%, *Actin-GAL4*: 15.34 ± 1.05%; *UAS-Hsp70B*: 19.04 ± 1.87%, and *Da-GAL4*: 14.88 ± 1.43%) (Fig. 3B).

Overall, these results strongly suggest that *Hsp70* genes are responsible for the ability of infected animals to move to high temperatures and that Hsp70 induction is important for stimulating the behavioral fever response.

### Behavioral fever results in the death of parasitoid wasps and is beneficial for fruit fly hosts

**Fig. 4.**
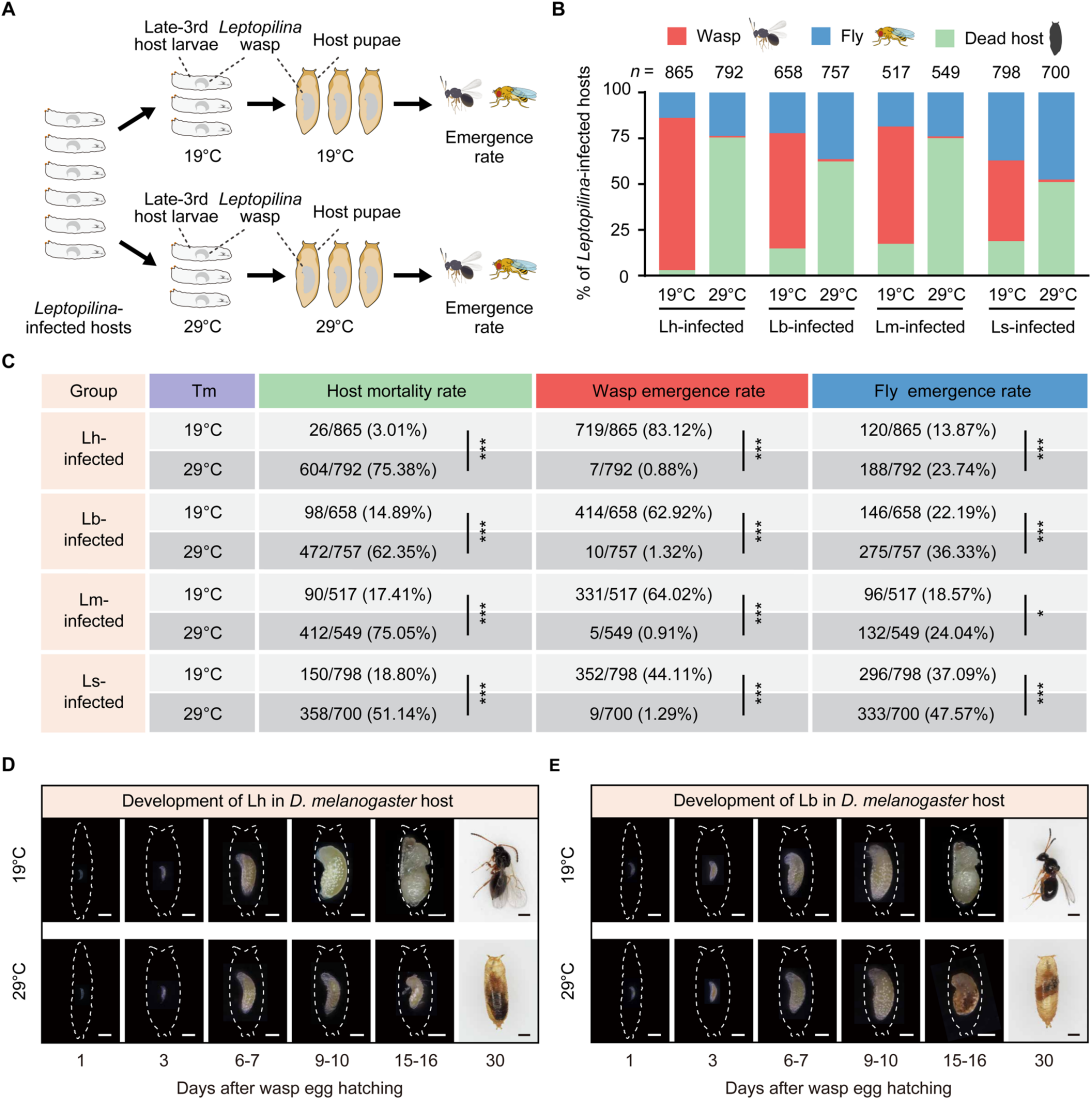
Behavioral fever is beneficial for fly hosts. (**A**) Schematic diagram of the emergence rate experiment for *Leptopilina*-infected *D. melanogaster* hosts at 19° and 29°C. (**B** and **C**) Analysis of the wasp emergence rate, fly emergence rate, and host mortality rate of *D. melanogaster* hosts infected by *L. heterotoma* (Lh-infected), *L. boulardi* (Lb-infected), *L. myrica* (Lm-infected), and *L. syphax* (Ls-infected) at 19° and 29°C. Significance was determined by Fisher’s exact test (*P < 0.05; ***P < 0.001). (**D** and **E**) Representative images of different developmental statuses of *L. heterotoma* (D) and *L. boulardi* (E) in *D. melanogaster* hosts at 19° and 29°C. The outline in each representative image refers to the host larval epidermis or host pupal case. Scale bars, 500 μm.

We split infected *D. melanogaster* host larvae into two groups and raised them at 19° and 29°C to determine whether behavioral fever benefits host in defending against parasitoid wasp infection (Fig. 4A). We found that the mortality of infected hosts was remarkably low at 19°C, and most of these infected hosts ultimately developed into parasitoid wasp adults, with a smaller fraction emerging as *D. melanogaster* adults (Fig. 4, B and C). However, the mortality of infected hosts increased significantly at 29°C (*L. heterotoma*–infected host larvae: 75.38% at 29°C and 3.01% at 19°C; *L. boulardi*–infected host larvae: 62.35% at 29°C and 14.89% at 19°C; *L. myrica*–infected host larvae: 75.05% at 29°C and 17.41% at 19°C; *L. syphax*–infected host larvae: 51.14% at 29°C and 18.80% at 19°C) (Fig. 4, B and C). Similarly, we found that the emergence rate of *Leptopilina* wasps in infected hosts at 29°C was significantly lower than that of *Leptopilina* wasps at 19°C (*L. heterotoma*–infected host larvae: 0.88% at 29°C and 83.12% at 19°C; *L. boulardi*–infected host larvae: 1.32% at 29°C and 62.92% at 19°C; *L. myrica*–infected host larvae: 0.91% at 29°C and 64.02% at 19°C; *L. syphax*–infected host larvae: 1.29% at 29°C and 44.11% at 19°C) (Fig. 4, B and C). Notably, nearly all the infected *D. melanogaster* hosts (wasp-host mixture) died at the host pupal stage at 29°C. We then dissected the infected host pupae and found that the development of *Leptopilina* wasp larvae was delayed, especially at 9 to 10 days after egg hatching (Fig. 4, D and E). *Leptopilina* wasp larvae in infected hosts subsequently died at 15 to 16 days after wasp egg hatching (Fig. 4, D and E). As a result, the emergence rate of infected *D. melanogaster* hosts at 29°C was significantly greater than that of *D. melanogaster* hosts at 19°C (*L. heterotoma*–infected host larvae: 23.74% at 29°C and 13.87% at 19°C; *L. boulardi*–infected host larvae: 36.33% at 29°C and 22.19% at 19°C; *L. myrica*–infected host larvae: 24.04% at 29°C and 18.57% at 19°C; *L. syphax*–infected host larvae: 47.57% at 29°C and 37.09% at 19°C) (Fig. 4, B and C). These results showed that a “hot” temperature could help kill the infecting parasitoid wasps and benefit host survival, indicating that behavioral fever is an adaptive strategy for *D. melanogaster* hosts to fight parasitoid wasp infection.

### Behavioral fever strengthens the immunity of infected fly hosts

**Fig. 5.**
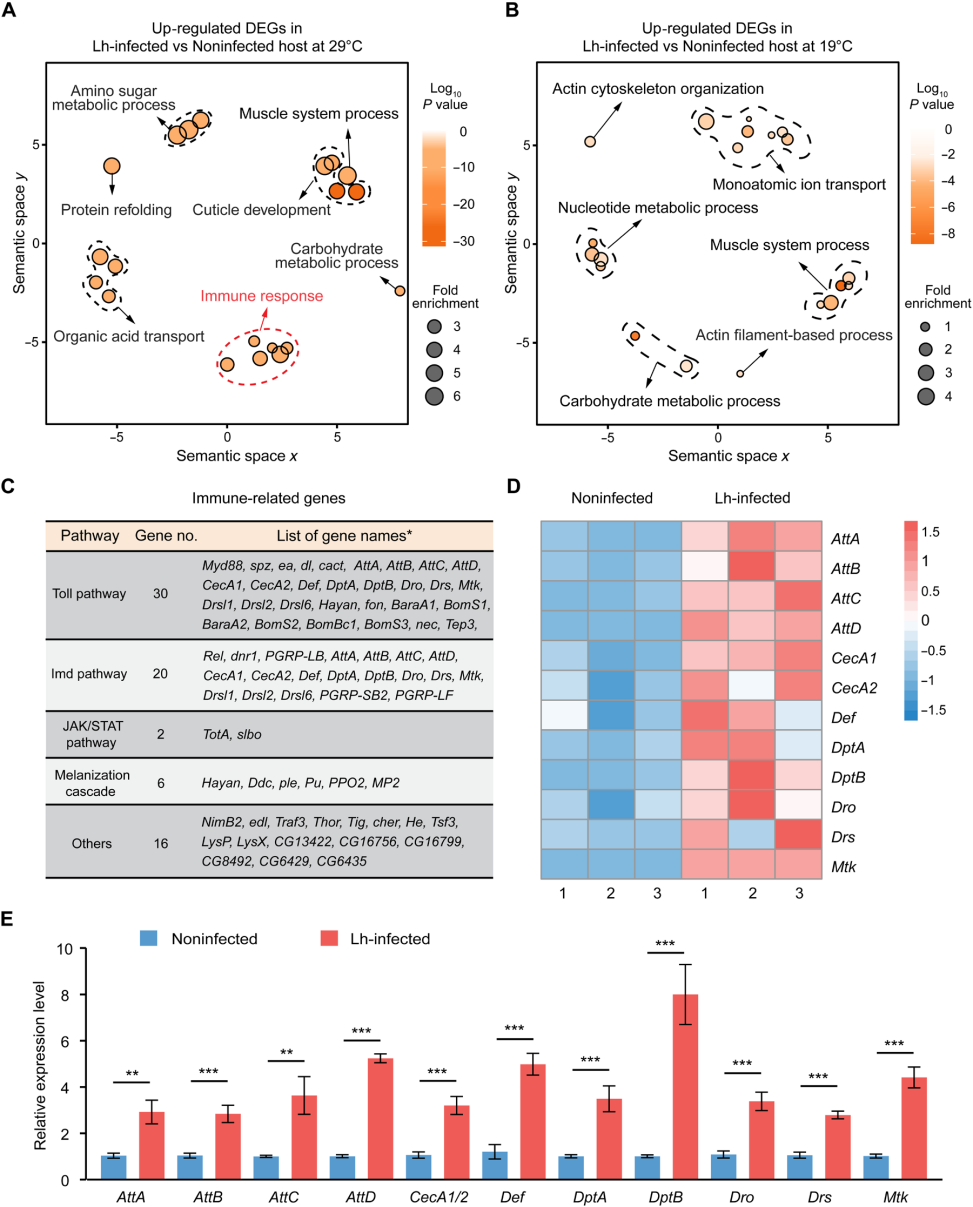
Behavioral fever induces the expression of many immune genes in infected fly hosts. (**A**) GO enrichment analysis of up-regulated DEGs and a comparison with the transcriptome data for Lh-infected and noninfected hosts at 29°C. Lh-infected, *L. heterotoma*-infected 1-day-old host pupae; Noninfected, noninfected 1-day-old host pupae. (**B**) GO enrichment analysis of up-regulated DEGs and a comparison with the transcriptomic data for Lh-infected and noninfected hosts at 19°C. (**C**) Functional categories of 58 up-regulated immune genes in Lh-infected *D. melanogaster* hosts at 29°C. The downstream *APM* genes were included in Toll and Imd pathways, respectively. (**D**) Expression profiles of *AMP* genes in the transcriptome data of Lh-infected and noninfected hosts at 29°C. The red and blue colors represent high to low expression levels, respectively, based on the FPKM value. (**E**) Relative mRNA levels of *AMP* genes in Lh-infected *D. melanogaster* 1-day-old pupae at 29°C determined using qPCR. Noninfected 1-day-old host pupae were used as controls. The pair of primers used for *CecA1* and *CecA2* qPCR analysis was the same. The experiments were performed with seven biological replicates. The data are presented as the means ± SEMs. Significance was determined by two-tailed unpaired Student’s *t* test (**P < 0.01; ***P < 0.001).

The following question then arises: How do *D. melanogaster* hosts acquire adaptive traits against parasitoid wasp infection at “hot” temperatures? We answered this question by generating transcriptome data from noninfected and *L. heterotoma*–infected *D. melanogaster* hosts raised at 19° and 29°C, respectively. The wasp larvae grow slowly during the host larval stage, begin to massively consume nutrients, and grow rapidly in the host pupal stage. Therefore, we chose host samples at the 1-day-old pupal stage for the transcriptomic experiments. The analysis of differentially expressed genes (DEGs) between *L. heterotoma*–infected hosts and noninfected controls revealed a number of significantly up-regulated genes and down-regulated genes (456 up-regulated genes in *L. heterotoma*–infected versus noninfected hosts at 19°C; 1443 up-regulated genes in *L. heterotoma*–infected versus noninfected hosts at 29°C; 1277 down-regulated genes in *L. heterotoma*–infected versus noninfected hosts at 19°C; and 1801 down-regulated genes in *L. heterotoma*–infected versus noninfected hosts at 29°C) (fig. S10). Gene Ontology (GO) analysis revealed several enriched functional terms among the up-regulated genes in *L. heterotoma*–infected hosts at 29°C, including the immune response, cuticle development, protein refolding, muscle system process, amino sugar metabolic process, carbohydrate metabolic process, and organic acid transport (Fig. 5A and table S3). However, no immune response genes were enriched in up-regulated genes in *L. heterotoma*–infected hosts at 19°C (Fig. 5B and table S4). *D. melanogaster* hosts have ~245 immune genes (*32*). Among them, 58 immune genes associated with the Toll pathway (*33*–*36*), the Imd pathway (*37*, *38*), the Jak/Stat pathway (*39*, *40*), the melanization pathway (*41*, *42*), and other pathways were significantly up-regulated in *L. heterotoma*–infected hosts at 29°C (Fig. 5C and table S5). These results suggest that behavioral fever helps strengthen the immune response of infected hosts, thereby resulting in substantial mortality of parasitoid wasps and an increase in host survival rates.

### An increase in host antimicrobial peptide levels causes parasitoid wasp death

**Fig. 6.**
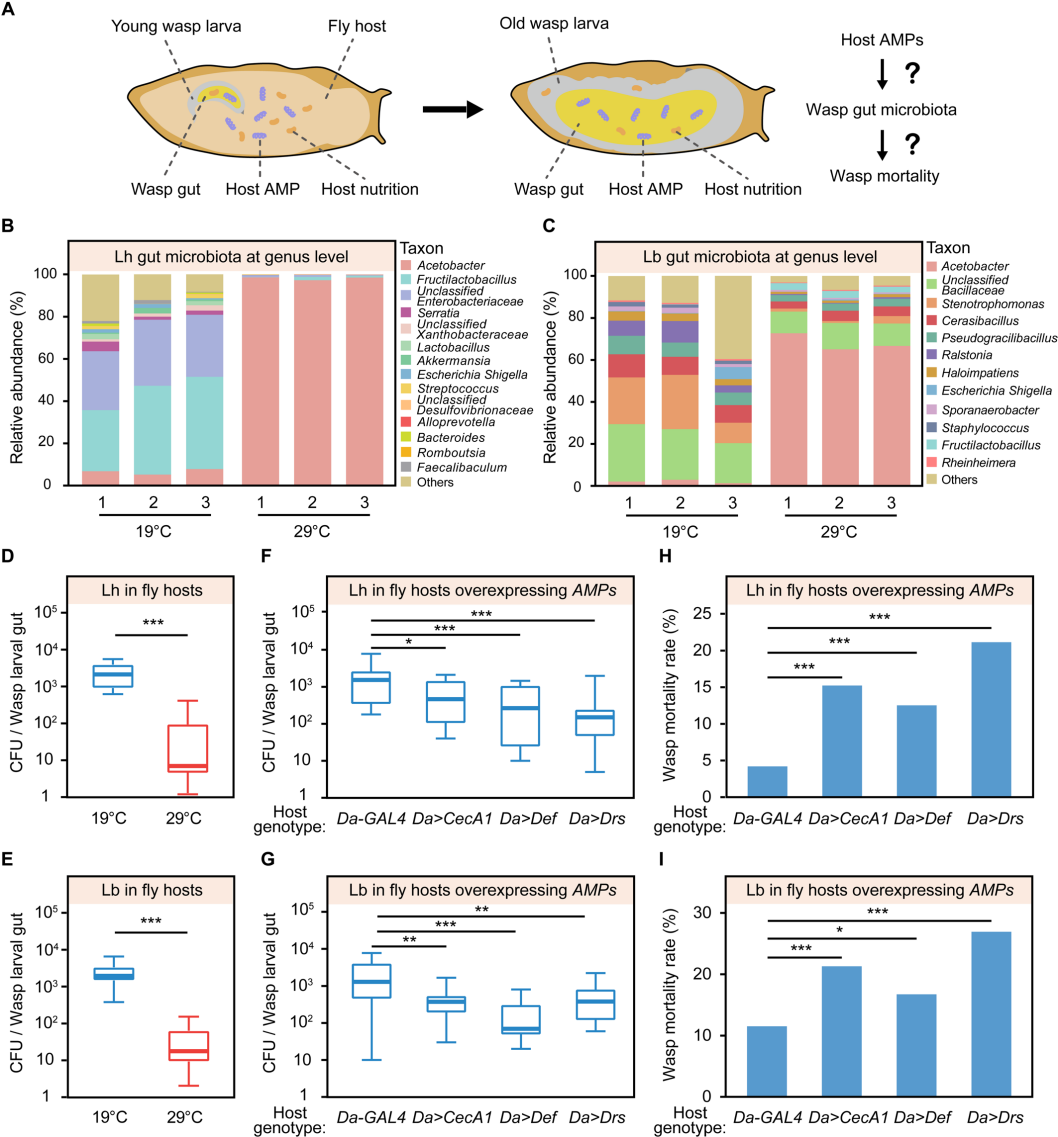
Increased levels of host AMPs cause wasp gut microbiota dysbiosis. (**A**) Schematic diagram of host AMPs impairing parasitoid wasp gut microbiota and mortality. (**B** and **C**) Bacterial community composition at the genus level of the gut microbiota in *L. heterotoma* larvae (B) and *L. boulardi* larvae (C) at 19° and 29°C. (**D** and **E**) CFUs of the gut microbiota in *L. heterotoma* larvae (D) and *L. boulardi* larvae (E) at 19° and 29°C. The experiments were performed with 26 biological replicates. Significance was determined by the Mann-Whitney *U* test (***P < 0.001). (**F** and **G**) CFUs of gut bacteria in *L. heterotoma* larvae (F) and *L. boulardi* larvae (G) living in different hosts overexpressing AMP genes (*CecA1*, *Def*, and *Drs*). The host genotypes are *Da-GAL4* (control), *Da>CecA1*, *Da>Def*, and *Da>Drs*. The experiments were performed with at least 22 biological replicates. Significance was determined by the Kruskal-Wallis test with Dunn’s multiple comparisons test (*P < 0.05; **P < 0.01; ***P < 0.001). (**H** and **I**) Mortality rates of *L. heterotoma* (H) and *L. boulardi* wasps (I) living in different hosts overexpressing AMP genes. Left to right in (H): *n* = 500, 578, 455, and 497 hosts; left to right in (I): *n* = 477, 502, 496, and 360 hosts. Significance was determined by Fisher’s exact test (*P < 0.05; ***P < 0.001).

*D. melanogaster* hosts have a total of 14 classic *AMP* genes, including 4 *Attacin* genes (*AttA*, *AttB*, *AttC*, and *AttD*), 4 *Cecropin* genes (*CecA1*, *CecA2*, *CecB*, and *CecC*), 2 *Diptericin* genes (*DptA* and *DptB*), one *Defensin* gene (*Def*), one *Drosocin* gene (*Dro*), one *Drosomycin* gene (*Drs*), and one *Metchnikowin* gene (*Mtk*) (*43*). The above transcriptomic results revealed that the expression of at least 12 *AMP* genes (*AttA*, *AttB*, *AttC*, *AttD*, *CecA1*, *CecA2*, *DptA*, *DptB*, *Def*, *Dro*, *Drs*, and *Mtk*) increased significantly in *L. heterotoma*–infected hosts at 29°C (Fig. 5D). qPCR analysis further showed that the expression of host *AMP* genes not only increased in *L. heterotoma*–infected hosts but also increased in *L. boulardi*–, *L. myrica*–, and *L. syphax*–infected *D. melanogaster* hosts at 29°C (Fig. 5E and fig. S11). The host is the only food resource for parasitoid larvae, and *Leptopilina* wasp larvae consume all nutrients in *D. melanogaster* host tissues for their own development, leading to the eventual transfer of increased levels of host antimicrobial peptides (AMPs) into the gut of parasitoid wasps (Fig. 6A). We then hypothesized that host AMPs could have adverse effects on the gut microbiota composition, diversity, and richness of parasitoid wasps, consequently resulting in high wasp mortality.

We tested whether increased host AMP levels induce parasitoid wasp gut microbiota dysbiosis by generating 16*S* ribosomal RNA (rRNA) sequencing libraries of the *Leptopilina* wasp larval gut microbiota (9 to 10 days after egg hatching) in infected hosts at 19° and 29°C. Compared with those of the 19°C group of each *Leptopilina* wasp species, the Shannon diversity index and Simpson diversity index, which account for species diversity and richness, were significantly reduced in the *Leptopilina* larvae at 29°C (fig. S12, A to H). Principal coordinate analysis (PCoA) results further revealed significant differences in the wasp gut microbial composition between the 19° and 29°C groups of each *Leptopilina* wasp species (fig. S12, I to J). Moreover, the distribution of the top 10 species at the genus level revealed significant differences in the intestinal microbial community between the 19° and 29°C groups of *Leptopilina* wasp larvae (Fig. 6, B and C, and fig. S12, M and N). For example, the dominant genera found in *L. heterotoma* larvae at 19°C were *Fructilactobacillus* (38.20%), unclassified Enterobacteriaceae (29.52%), *Acetobacter* (6.63%), *Serratia* (2.65%), and unclassified Xanthobacteraceae (1.71%); however, the dominant genera found in *L. heterotoma* larvae at 29°C were *Acetobacter* (98.15%), *Fructilactobacillus* (0.79%), unclassified Enterobacteriaceae (0.71%), unclassified Desulfovibrionaceae (0.07%), and *Escherichia Shigella* (0.05%) (Fig. 6B). We subsequently performed qPCR to quantify the number of bacteria in the gut of parasitoid wasp larvae at the two different temperatures. A marked decrease in the number of gut bacteria was detected in all four *Leptopilina* wasp larvae in *D. melanogaster* hosts at 29°C compared with *Leptopilina* wasp larvae at 19°C (fig. S13). The reduction in gut bacteria at 29°C was confirmed by plating wasp gut homogenates onto MRS medium agar plates and counting the number of colony-forming units (CFUs) (Fig. 6, D and E, and fig. S14).

We tested the effects of excessive expression of *AMP* genes (e.g., *CecA1*, *Def*, and *Drs*) in *D. melanogaster* hosts using the ubiquitous *Da-GAL4* line (fig. S15) to provide direct and strong evidence that the induction of *D. melanogaster* host AMPs at 29°C is the cause of wasp gut microbiota dysbiosis and subsequent wasp death. The results revealed that the number of CFUs of the gut microbiota of *L. heterotoma* and *L. boulardi* wasps in the *Da*>*CecA1*, *Da*>*Def*, and *Da*>*Drs* hosts was significantly lower than that in the gut microbiota of the *Da-GAL4* control groups (Fig. 6, F and G). In addition, we observed markedly elevated mortality rates of *Leptopilina* parasitoid wasps in hosts overexpressing *CecA1*, *Def*, and *Drs* AMPs (Fig. 6, H and I).

Collectively, these results indicate that increased levels of host AMPs cause wasp gut microbiota dysbiosis, which, in turn, leads to the mortality of parasitoid wasps in *D. melanogaster* hosts at a “hot” temperature.

## DISCUSSION

Behavioral fever, defined as an acute change in thermal preference in response to parasite infection, was first reported in desert iguana about half a century ago (*6*). Since then, this fascinating phenomenon has been reported in other ectothermic animals when they are infected by microparasites (*9*, *11*, *44*, *45*). Here, we found that *D. melanogaster* larvae exploit behavioral fever as a defense strategy against parasitoid wasp infection. To the best of our knowledge, this study shows that ectothermic animals exhibit behavioral fever when infected by parasitoid wasps, a large group of macroparasites, a phenomenon not previously described.

In endothermic animals, prostaglandin E2 (PGE2) produced by brain vascular endothelial cells has been found to be a major pyrogenic mediator of the fever response (*46*, *47*). However, limited information is available regarding the mediators of behavioral fever in ectothermic animals. The *Hsp70* gene family is highly conserved across animals and serves as a group of molecular chaperones to maintain protein homeostasis (*48*). Increasing evidence indicates that *Hsp70* genes might play essential roles in innate immunity, helping host animals defend against parasitic infection (*49*, *50*). Our functional results revealed that the *Hsp70* gene family is also essential for the behavioral immune response in infected *D. melanogaster* hosts. Namely, parasite infection triggers increased expression of *Hsp70* in hosts, which, in turn, elicits a behavioral fever response. Notably, increased expression of *Hsp70* genes was widely observed in other ectothermic vertebrates and ectothermic invertebrates when they were infected by different parasites (e.g., bacteria, fungi, viruses, and parasitic insects), which is very similar to our results in parasitized *D. melanogaster* hosts (table S6). For example, the expression of *Hsp70* genes was up-regulated in largemouth black bass and zebrafish when they were infected by *A. hydrophila* or a simulated virus, respectively (*10*, *51*). Similarly, both infected largemouth black bass and zebrafish exhibit a behavioral fever response (*9*, *10*). As such, the *Hsp70* gene family is likely a common regulator that mediates behavioral fever responses in infected ectothermic animals. Because known thermal preference genes are not involved in this behavioral response, the mechanism by which *Hsp70* contributes to behavioral fever remains unclear. Specifically, understanding how Hsp70 induction alters neuroendocrine signaling to drive changes in thermal preference behavior, as well as how it directly or indirectly regulates the expression of immune genes, represents critical and fascinating areas for future research.

These changes in the thermal regime have been suggested, but not experimentally confirmed, to favor the immune response and thus promote host survival. Our results demonstrate that a temperature of 29°C helps induce the significant up-regulation of many immune genes, including *Prophenoloxidase 2* (*PPO2*), *Dopa decarboxylase* (*Ddc*), *Thioester-containing protein 3* (*Tep3*), and 12 *AMP* genes, in infected *D. melanogaster* hosts. The *PPO* and *Ddc* genes contribute to the melanization reaction during the encapsulation process to protect against the eggs of parasitoid wasps (*52*, *53*). *TEP* family genes have been shown to participate in host defenses against parasitoid wasp infection (*54*). In agreement with these previous findings, we detected a significant increase in the survival rates of infected hosts when they were living at a high temperature. Notably, we detected high mortality of parasitoid wasp larvae in infected hosts. Our findings further revealed that behavioral fever can alter the composition and abundance of the gut microbiota in parasitoid wasp larvae, possibly through the consumption of large amounts of host AMPs. The gut microbiota plays a crucial role in the nutritional metabolism of insect hosts, and disturbances in the gut microbiota can impair insect growth and survival (*55*–*57*). Because AMPs are necessary for insect gut immunity to control the commensal community (*58*, *59*), excess AMPs cause gut microbiota dysbiosis and substantial mortality in parasitoid wasps. As such, this strategy could substantially reduce the wasp population and, in turn, benefit hosts.

**Fig. 7.**
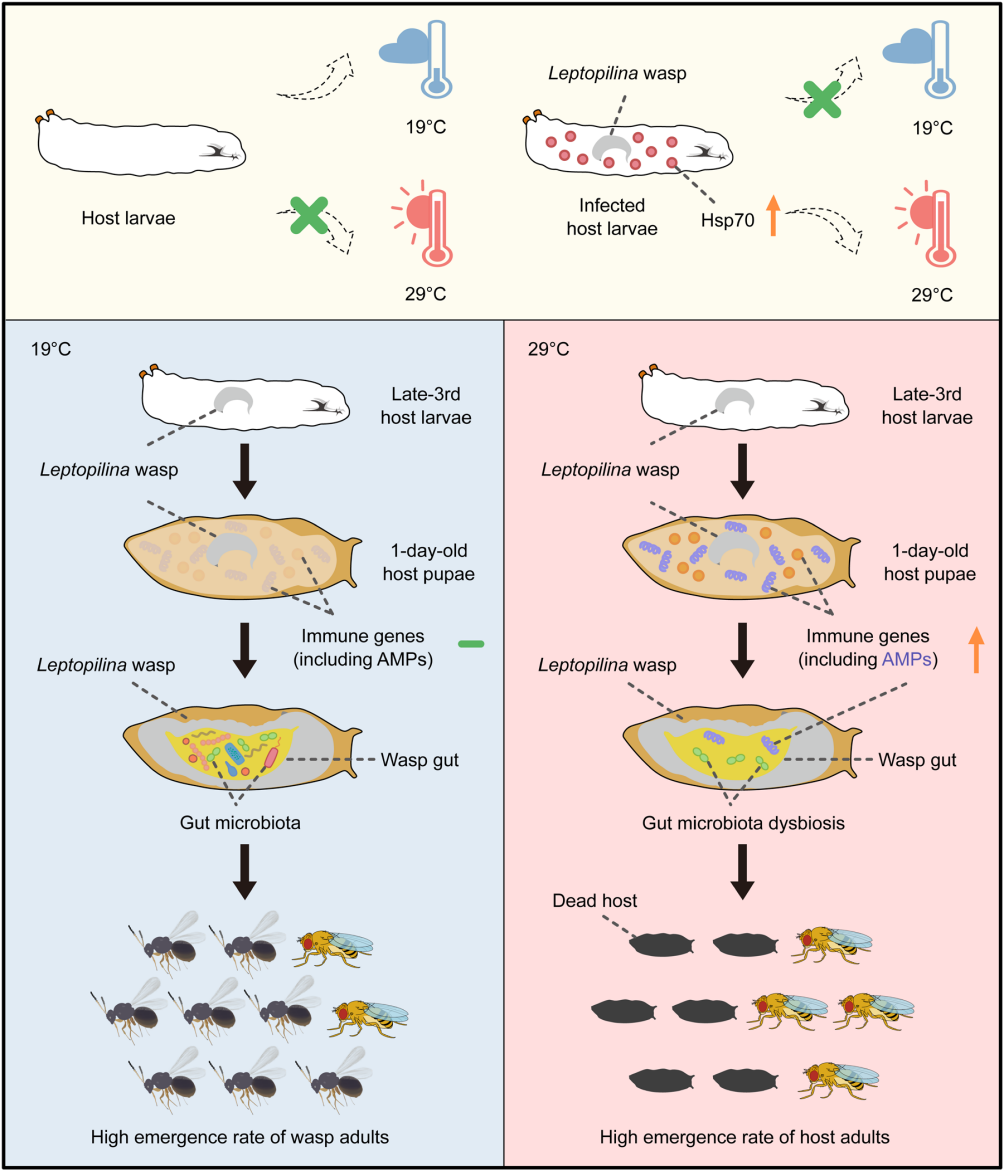
Proposed model for behavioral fever and the underlying mechanisms. The increased expression of *Hsp70* genes plays an important role in triggering the behavioral fever of infected fly hosts to fight parasitoid wasp infection. At the noninfected hosts’ preferred temperature (19°C), many *Leptopilina* wasp larvae overcome host immunity and successfully develop into adult wasps. Accordingly, the emergence rate of *D. melanogaster* hosts is relatively low. However, behavioral fever leads infected hosts to choose a hot temperature (29°C), which induces the expression of numerous immune genes, including AMPs. The excess host AMPs cause wasp gut microbiota dysbiosis and substantial wasp mortality, which, in turn, benefits host survival.

In summary, our study highlights the critical importance of behavioral fever in ectothermic animals to fight macroparasite infection and demonstrates the key role of *Hsp70* induction in triggering behavioral fever and orchestrating the immune response to benefit host animals (Fig. 7).

## MATERIALS AND METHODS

### Insects

*L. heterotoma* and *L. boulardi* were provided by I. Andó (HUN-REN Biological Research Centre) and D. Hultmark (Umea University), respectively. Local parasitoid wasp species of *L. myrica* and *L. syphax* were collected from Taizhou (28°50′N, 120°34′E), Zhejiang, China, in 2021 and 2018, respectively. All parasitoid wasp species were maintained in *D. melanogaster* (*w^1118^*) as regular hosts. The newly emerged wasp adults were provided with apple juice agar media until they were exposed to the fly hosts. The apple juice agar recipe included 27 g of agar, 33 g of brown sugar, and 330 ml of pure apple juice in 1000 ml of diluted water.

*D. melanogaster* strains *w^1118^* (BL5905), *Hsp70A^−^B^−^* (BL8841), and *Hsp70A^−^* (BL8842) were obtained from the Bloomington *Drosophila* Stock Center (BL). *UAS-Hsp70RNAi* (THU1287) and the ubiquitous *GAL4* line *Da-GAL4* (TB00103) were obtained from the Tsinghua Fly Center. Another ubiquitous *GAL4* line, *Actin-GAL4*, was provided by D. Kalderon (Columbia University) (*60*). The *UAS-CecA1*, *UAS-Def*, and *UAS-Drs* lines were provided by Z. Zhai (Hunan Normal University) (*61*). The coding regions of *Hsp70Aa* and *Hsp70Bb* were amplified from the cDNA of *D. melanogaster* using the primers listed in table S7 to generate the *UAS-Hsp70A* and *UAS-Hsp70B* transgenic lines. The resulting cDNA fragment was digested with Kpn-I (NEB) and inserted into the pUAST-attB vector. The plasmids encoding *Hsp70A* and *Hsp70B* were injected into *D. melanogaster* embryos and integrated into the attP sites on chromosome 2 (cytological locus 25C6) and chromosome 3 (cytological locus 86F8), respectively. *D. melanogaster* were reared on standard cornmeal/molasses/agar media (*62*) at 25°C.

### Preparing *D. melanogaster* host larvae for thermal preference assays

The procedure for preparing *D. melanogaster* host larvae for thermal preference experiments was performed as described in a previous study, with minor modifications (*23*). We allowed the adult flies to recover from CO_2_ for ≥48 hours in vials containing fly food with yeast paste to increase egg production. Each vial contained 100 females and 60 males (5 to 15 days old) to facilitate egg laying for a period of 2 hours, and the animals were aged. *L. heterotoma*, *L. boulardi*, and *L. myrica* female wasps were allowed to infect second instar (60 hours AEL) larvae, and *L. syphax* female wasps were allowed to infect first instar (30 hours AEL) *D. melanogaster* host larvae. This difference in the developmental stages of *Drosophila* host larvae used for infection is due to *L. syphax* being less effective at parasitizing second instar larvae and more successful in infecting first instar larvae. The parasite-to-host ratio for each *Leptopilina* species was 1:10, and the infection time was 2 hours. We randomly selected 50 larvae from each experimental group immediately after infection and dissected them to detect the presence of wasp eggs, thereby confirming the infection status. Only groups with an infection rate exceeding 95% were used for subsequent thermal preference experiments. The infection rate was calculated using the following formula: (number of infected host larvae/total dissected host larvae) × 100%. To simulate the mechanical stress of parasitoid wasp ovipositor puncture, sterilized minutien pins with a 0.0125-mm tip diameter (Fine Science Tools) were used to prick second instar larvae for thermal preference experiments.

Once the *Leptopilina*-infected or noninfected host larvae reached the early-third instar (72 hours AEL) or the late-third instar (120 hours AEL) stages, we scooped out the fly food containing the host larvae into a 15% sucrose solution in 50-ml tubes to allow the host larvae to float and the food debris to sink. After a brief incubation, the top layer containing the host larvae was repeatedly transferred to another 50-ml tube containing a fresh 15% sucrose solution until all remaining food debris was removed (usually two to three times). The host larvae subsequently underwent two additional washes with ddH_2_O to eliminate sucrose and other debris. The collected host larvae were incubated for 15 to 30 min at room temperature in a 90-mm dish under dim light with adequate moisture to prevent desiccation before being used for the assays.

### Temperature gradient assays

The apparatus for performing the temperature gradient assays included a dry bath incubator (Miulab) and an aluminum plate (18 cm in length, 10.8 cm in width, and 0.7 cm in height). The aluminum plate was divided into three parts: The left 2-cm region was connected to a cooling dry bath incubator, the right 2-cm region was connected to a heating dry bath incubator, and the remaining 14-cm region was filled with 2% agarose. The incubators were set at two different temperatures, which varied depending on the ambient temperature, to establish a temperature gradient ranging from 19° to 31°C. We gently sprayed the gels with water as needed to prevent them from drying. We verified the temperature gradient by monitoring the surface temperatures on the test plates in each of seven zones (2 cm) using a thermometer (Testo).

Once the temperature gradient on the test plate stabilized, 100 to 150 prepared *Leptopilina*-infected or noninfected host larvae were placed in the central zone of the plate, which was maintained at 25°C (release zone). After a 15-min interval, photographs of the test plate were captured, and the number of larvae in each zone was counted. The thermal preference was calculated using the following formula: (number of larvae in a given zone/total number of larvae in all zones) × 100%.

### Thermal two-way choice assays

We used an aluminum plate (18 cm in length, 10.8 cm in width, and 0.7 cm in height) filled with 2% agarose to prepare plates for the thermal two-way choice assay. The plate was placed on top of two adjacent aluminum blocks, which were individually temperature controlled using a dry bath incubator (Miulab). The surface temperatures at the center of each side were monitored using a thermometer (Testo), which was 19°C on one side and 29°C on the other. We gently sprayed the gels with water as needed to prevent them from drying.

Thermal two-way choice assays were initiated by placing 100 to 150 prepared *D. melanogaster* host larvae in a line at the border between the 19° and 29°C sides (release zone). After a 15-min interval, photographs of the test plate were captured, and the number of larvae on the 19° or 29°C side was counted. The thermal preference was calculated using the following formula: (number of larvae on one side/total number of larvae on both sides) × 100%.

### Quantitative real-time PCR

Total RNA was extracted from different *D. melanogaster* at different developmental stages (late-third instar larval stage and 1-day-old pupal stage) using the RNeasy Mini Kit (Qiagen) and then reverse transcribed into cDNA using HiScript III RT SuperMix for qPCR (Vazyme) according to the manufacturer’s protocol. qPCR was performed in the QuantStudio3 Real-Time PCR System (Thermo Fisher Scientific) with the ChamQ SYBR qPCR Master Mix Kit (Vazyme) under the following temperature cycling conditions: 30 s at 95°C, followed by 45 cycles of three-step PCR for 10 s at 95°C, 20 s at 55°C, and 20 s at 72°C. The RNA levels of the target genes were normalized to that of the *Actin-5C* mRNA, and the relative concentration was determined using the 2^−ΔΔCt^ method. All primers used for qPCR in this study are listed in table S7.

### Transcriptome sequencing and analysis

*Leptopilina*-infected and noninfected hosts at different stages of development were sampled for the transcriptomic analysis. Total RNA was extracted from each sample using the RNeasy MiniKit (Qiagen). Construction of the cDNA library and paired-end RNAseq (Illumina, NovaSeq 6000) were performed by Berry Genomics Co. Ltd.

Fastp v0.23.4 (*63*) was used to remove low-quality reads and adapter sequences. Clean reads were mapped to the reference *D. melanogaster* genome (http://flybase.org/) using HISAT2 v2.0.5 (*64*). The number of reads mapped to each gene was determined via StringTie v2.2.0 (*65*). The differential expression analysis of read counts was conducted using DESeq2 v1.42.1 (*66*). An absolute value of log_2_ (fold change) ≥ 1 and an adjusted *P* value of <0.05 were set as the criteria for DEGs in late-third instar host larvae, and an absolute value of log_2_ (fold change) ≥ 1.2 and an adjusted *P* value <0.05 were set as the criteria for DEGs in 1-day-old host pupae. Fragments per kilobase of exon model per million mapped fragments (FPKM) values were calculated using StringTie v2.2.0 and normalized to mitigate the effects of the gene length and sequencing depth on read counts, followed by presentation in the form of an expression profile heatmap. GO enrichment analysis of DEGs was conducted using clusterProfiler v4.12.0 (*67*). The significantly enriched GO terms were summarized and visualized using REVIGO v20150217beta (*68*).

### Sequence analysis of *Hsp70* genes

The coding sequences and amino acid sequences of different *Hsp70* genes were obtained from FlyBase (http://flybase.org/). The phylogenetic tree was constructed using IQ-TREE v2.1.3 (*69*), and a sequence similarity analysis was performed using BLAST at NCBI (https://ncbi.nlm.nih.gov/). Multiple sequence alignments were visually displayed with Jalview v2.11.4.0 (*70*).

### Western blot analysis

Total proteins were extracted from 20 *D. melanogaster* host larvae with a Minute Total Protein Extraction Kit for Insects (Invent) for Western blotting according to the manufacturer’s protocol. Three replicates were performed for each group. The proteins were quantified with a BCA protein assay kit (Thermo Fisher Scientific), and the same amount of protein was reserved for the SDS–polyacrylamide gel electrophoresis analysis. The proteins were subsequently transferred to polyvinylidene difluoride membranes (Millipore). The membranes were incubated in a blocking solution (Tris-buffered saline containing 0.1% Tween 20 and 2% bovine serum albumin) for 3 hours and probed overnight at 4°C with the primary antibodies. The primary antibodies used were anti-Hsp70 (3A3) (1:4000 dilution; Thermo Fisher Scientific, #MA3-006) and anti-actin (1:5000 dilution; ABclonal, #AC004). The horseradish peroxidase–conjugated goat anti-mouse IgG (H+L) secondary antibody (ABclonal, #AS003) was used at a dilution of 1:5000.

### The emergence rates of *D. melanogaster* and *Leptopilina* wasps in infected hosts

The infected host larvae (late-third instar, 120 hours AEL) were divided into two groups and maintained at 19° and 29°C to determine the emergence rates. The wasp emergence rate, fly emergence rate, and host mortality rate were calculated using the following formulas: Wasp emergence rate = (number of emerged wasps/number of total hosts) × 100%; Fly emergence rate = (number of emerged flies/number of total hosts) × 100%; Host mortality rate = (number of dead hosts/number of total hosts) × 100%.

### Observation of the parasitoid wasp developmental status

*L. heterotoma* and *L. boulardi* were dissected from infected *D. melanogaster* hosts, and their development status was monitored using the VHX-7000C imaging system (KEYENCE). Photos were taken at 1-day intervals after wasp egg hatching. All measurements were performed at least 20 randomly selected wasps. The outline around each representative image in Fig. 4 (D and E) refers to the larval epidermis or pupal case of *D. melanogaster* hosts.

### *Leptopilina* wasp gut microbiota analysis

*Leptopilina* wasp larvae were surface sterilized with ethanol, and the guts were dissected in sterile phosphate-buffered saline (PBS) on an ice plate under a stereoscope. A total of 40 wasp guts per replicate were sampled from *L. heterotoma*, *L. boulardi*, *L. myrica*, and *L. syphax* larvae at 19° and 29°C. Three replicates were performed for each sample. Bacterial DNA was extracted using the FastPure Cell/Tissue DNA isolation Mini Kit (Vazyme). A region encompassing the V3 and V4 hypervariable regions of the 16*S* rRNA gene was amplified using the primers 338F and 806R. The primers used are shown in table S7. The resulting amplicons were sequenced on the Illumina NovaSeq 6000 platform (Biomarker). The raw reads were filtered by Trimmomatic v0.33 (*71*), and the primer sequences were identified and removed by Cutadapt v1.9.1 (*72*) to generate high-quality reads. Sequence denoising was performed using DADA2 v1.20.0 (*73*). The sequences were clustered at the 97% similarity level using USEARCH v10.0 (*74*), with a filtering threshold set at 0.005% of the total sequence number for the operational taxonomic unit (OTU) analysis. Taxonomic annotation was processed by a Bayesian classifier based on the Silva v138 database (*75*) using RDP Classifier v2.2 (*76*). The Shannon diversity index and Simpson diversity index were calculated using Mothur v1.30 (*77*). PCoA of the Bray-Curtis data was performed using QIIME2 v2020.6 (*78*).

### Quantification of gut bacteria by qPCR

The relative abundance of gut bacteria in parasitoid wasp larvae was quantified using a quantitative detection method (*79*). Briefly, fifteen guts were sampled from parasitoid wasp larvae, and genomic DNA was extracted using the FastPure Cell/Tissue DNA Isolation Mini Kit (Vazyme). Three replicates were performed for each group. qPCR analysis was performed to quantify bacterial abundance using a pair of universal eubacteria primers amplifying the 16*S* rRNA fragment. qPCR was performed with the QuantStudio3 Real-Time PCR System (Thermo Fisher Scientific) and the ChamQ SYBR qPCR Master Mix Kit (Vazyme). The housekeeping *tubulin* gene of the parasitoid wasps served as an endogenous control. The primers used are shown in table S7.

### Quantification of culturable bacteria in the wasp gut

The culturable bacteria in parasitoid wasp guts were quantified using a CFU assay. Dissected guts were collected in sterile grinding tubes containing 200 μl of MRS liquid culture medium. Aliquots of 10 μl from each sample were plated onto MRS agar medium and incubated at 30°C for 3 days. The bacterial colonies were counted, and the CFUs per wasp larval gut were calculated. Each experiment was conducted with 20 to 30 biological replicates, with 10 guts collected per replicate.

### Statistical analysis

All the statistical analyses were performed using GraphPad Prism v8.0 (GraphPad Software) and SPSS 26 (IBM). The normality of the distribution of the data was tested using the Shapiro-Wilk test. The Bartlett chi-square test was used to test the homogeneity of variance of the data, which was consistent with a normal distribution. We used two-tailed unpaired Student’s *t* tests to determine the statistical significance of differences between two treatments when a parametric test was appropriate. We used the Mann-Whitney *U* test for experiments requiring a nonparametric statistical test. One-way analysis of variance (ANOVA) with Tukey’s multiple comparisons test were used to compare differences in the mean values between multiple groups when a parametric test was appropriate. The Kruskal-Wallis test with Dunn’s multiple comparisons test were used to compare differences in the mean values between multiple groups for experiments requiring a nonparametric statistical test. Fisher’s exact test was used to compare the wasp emergence rate, fly emergence rate, and host mortality rate. Details of the statistical analyses are provided in the figure legends, including how significance was defined and the statistical methods used. The data are presented as the means ± SEMs. Significance values are indicated as follows: *P < 0.05, **P < 0.01, and ***P < 0.001.
