# Supplementary material for: Fruit flies exploit behavioral fever as a defense strategy against parasitic insects
Source: Sci Adv. 2025 Jun 11;11(24):eadw0191. doi: 10.1126/sciadv.adw0191 (PMC12154181; doi:10.1126/sciadv.adw0191)
Supplement: Supplementary file 1 — Figs. S1 to S15 Legends for tables S1 to S7 [file sciadv.adw0191_sm.pdf]

Science Advances
AAAS

Supplementary Materials for

Fruit flies exploit behavioral fever as a defense strategy against parasitic insects

Yifeng Sheng *et al.*

Corresponding author: Xuexin Chen, xxchen@zju.edu.cn; Xing-Xing Shen, xingxingshen@zju.edu.cn; Jianhua Huang, jhhuang@zju.edu.cn

Sci. Adv. **11**, eadw0191 (2025)
DOI: 10.1126/sciadv.adw0191

The PDF file includes:

Figs. S1 to S15
Legends for tables S1 to S7

Other Supplementary Material for this manuscript includes the following:

Tables S1 to S7

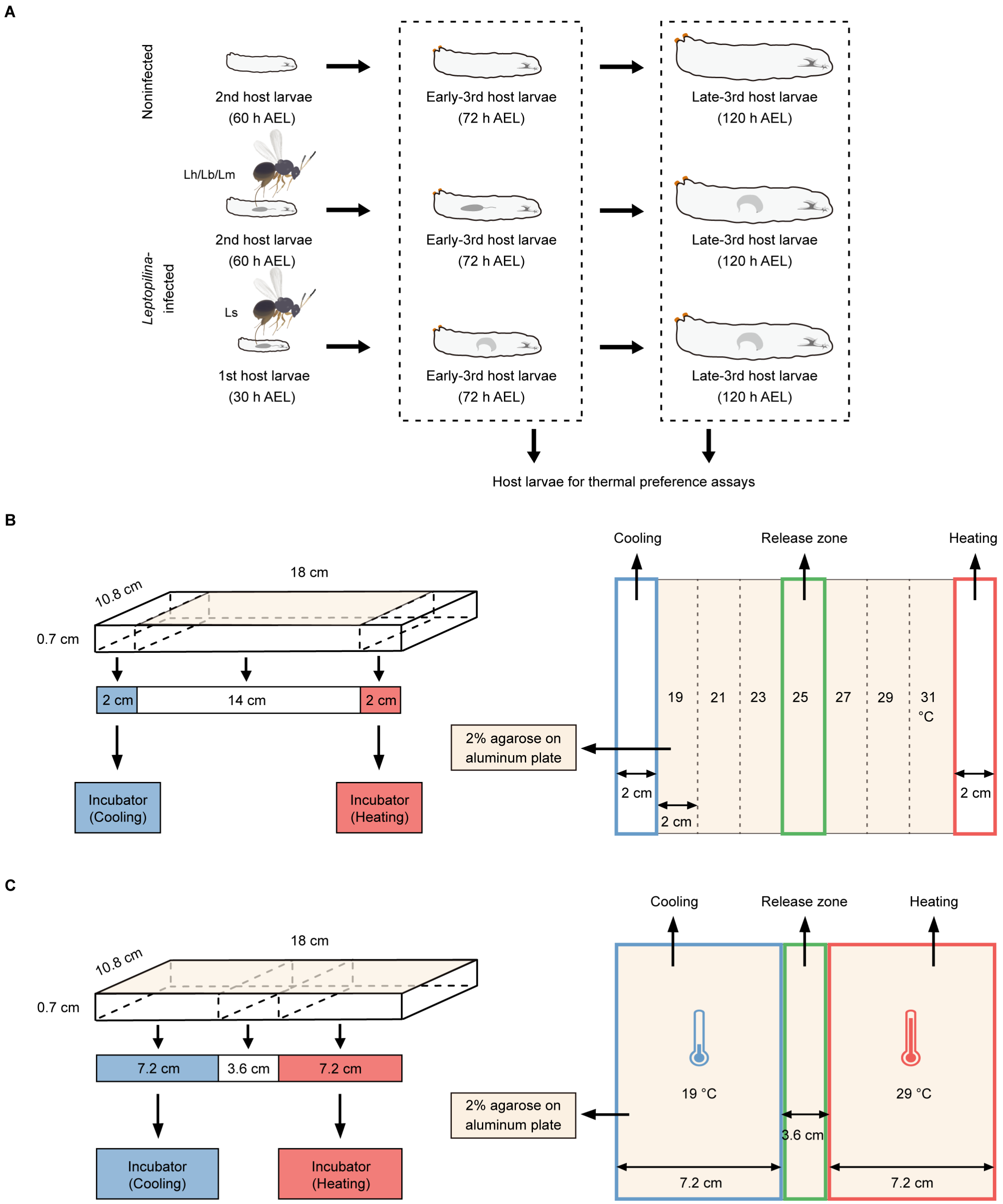

Fig. S1. Fruit fly host samples and apparatus for the thermal preference assays. (A) Preparation of host samples for the thermal preference assays. We used *L. heterotoma*, *L. boulardi*, and *L. myrica* female wasps to infect *Drosophila* 2nd instar larvae (60 h AEL) and *L. syphax* female wasps to infect *Drosophila* 1st instar larvae (30 h AEL). We subsequently collected early-3rd instar (72 h AEL) host larvae and late-3rd instar (120 h AEL) host larvae for the thermal preference assays. **(B)** Schematic diagram of the apparatus used for the temperature gradient assays. The aluminum plate was divided into three parts: the left 2 cm region was connected to a cooling

dry bath incubator, the right 2 cm region was connected to a heating dry bath incubator, and the remaining 14 cm region was filled with 2% agarose. The test plate (14 cm region) was divided into seven zones (2 cm each), with a temperature gradient ranging from 19 °C to 31 °C. The central zone of the plate was the host larva release zone. **(C)** Schematic diagram of the apparatus used for the two-way thermal choice assays. An aluminum plate filled with 2% agarose was placed on top of two adjacent aluminum blocks, which were individually temperature controlled using dry bath incubators. The surface temperature at the center of one side was 19 °C, and that at the other side was 29 °C. The central zone of the plate was the host larva release zone.

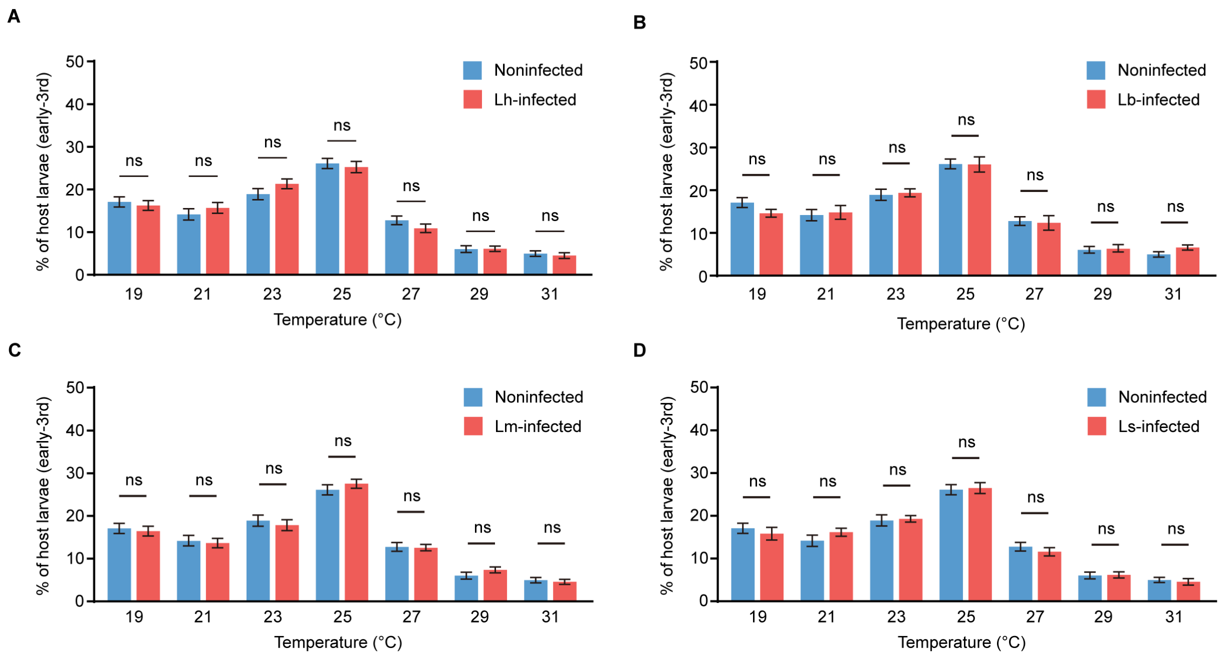

Fig. S2. Thermal preferences of *Leptopilina*-infected early-3rd instar host larvae. (A-D) Thermal preference of early-3rd instar *D. melanogaster* larvae infected by *L. heterotoma* (Lh-infected) (A), *L. boulardi* (Lb-infected) (B), *L. myrica* (Lm-infected) (C), or *L. syphax* (Ls-infected) (D). Noninfected early-3rd instar host larvae (noninfected) were used as controls. Experiments were performed with eight biological replicates. The data are presented as the means ± SEMs. Significance was determined by two-tailed unpaired Student's t test (ns: not significant).

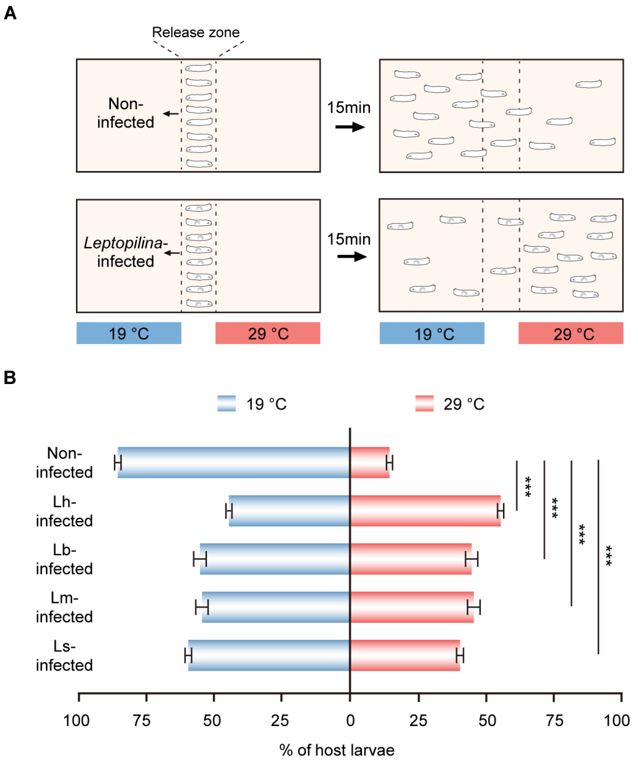

Fig. S3. *Leptopilina*-infected *D. melanogaster* host larvae prefer a high temperature. (A) Schematic diagram of the thermal two-way choice assay for *D. melanogaster* host larvae. Cold region: 19 °C; hot region: 29 °C. **(B)** The thermal preference of late-3rd instar host larvae infected by *L. heterotoma* (Lh-infected), *L. boulardi* (Lb-infected), *L. myrica* (Lm-infected), or *L. syphax* (Ls-infected). Noninfected late-3rd instar host larvae (noninfected) were used as controls. The experiments were performed with ten biological replicates. The data are presented as the means ± SEMs. Significance was determined by one-way ANOVA with Tukey's multiple comparison test (***: $p < 0.001$).

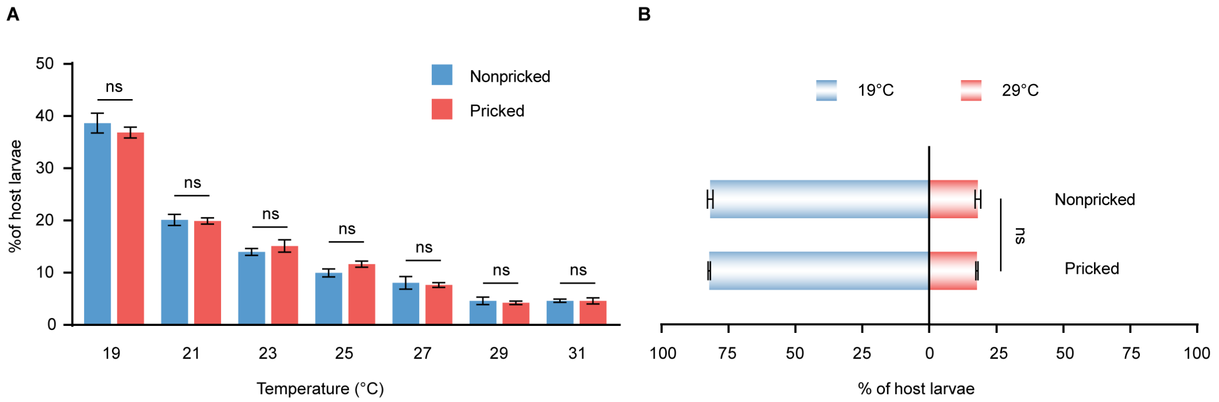

Fig. S4. Thermal preferences of pricked *D. melanogaster* host larvae. (A) Thermal preference of pricked late-3rd instar *D. melanogaster* host larvae assessed using a temperature gradient assay. **(B)** Thermal preference of pricked late-3rd instar *D. melanogaster* host larvae assessed using a thermal two-way choice assay. The nonpricked late-3rd instar host larvae were used as controls. The experiments were performed with ten biological replicates. The data are presented as the means ± SEMs. Significance was determined by two-tailed unpaired Student's t test (ns: not significant).

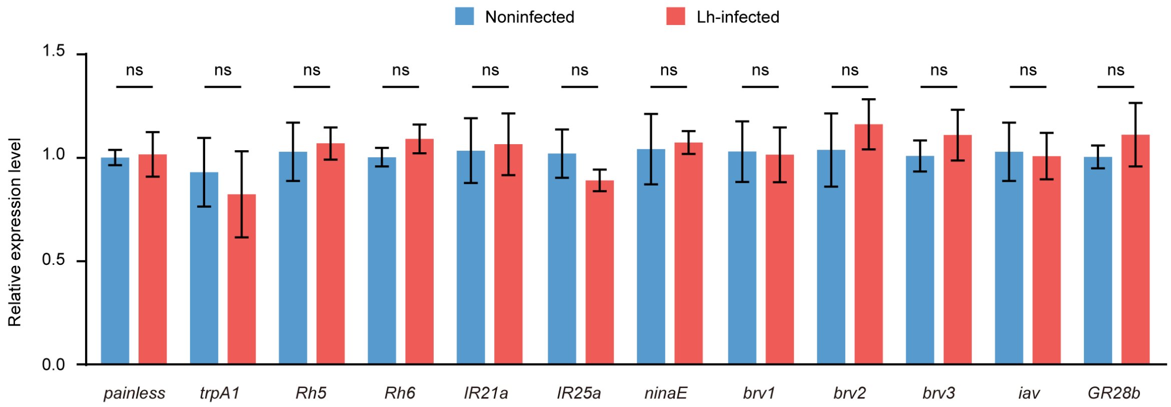

Fig. S5. The expression levels of renowned regulatory genes related to thermal preference. Relative mRNA levels of thermal preference regulatory genes, including *painless*, *trpA1*, *Rh5*, *Rh6*, *IR21a*, *IR25a*, *ninaE*, *brv1*, *brv2*, *brv3*, *iav* and *GR28b*. Lh-infected: late-3rd instar host larvae that were infected by *L. heterotoma*; Noninfected: noninfected late-3rd instar host larvae. The experiments were performed with 4 biological replicates. The data are presented as the means ± SEMs. Significance was determined by two-tailed unpaired Student's t test (ns: not significant).

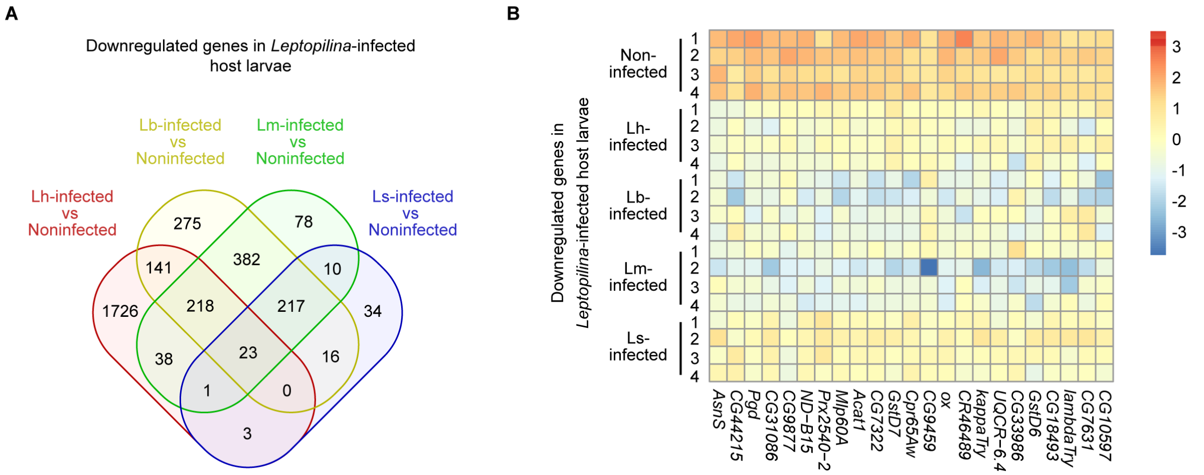

Fig. S6. Downregulated DEGs in infected host larvae. (A) Venn diagram illustrating the downregulated DEGs identified via a transcriptomic analysis of *Leptopilina*-infected late-3rd instar host larvae compared with noninfected late-3rd instar host larvae. Each circle represents a comparison, with the overlaps indicating common downregulated DEGs among different comparisons. Noninfected: noninfected host larvae; Lh-infected: *L. heterotoma*-infected host larvae; Lb-infected: *L. boulardi*-infected host larvae; Lm-infected: *L. myrica*-infected host larvae; Ls-infected: *L. syphax*-infected host larvae. **(B)** Expression profiles of downregulated DEGs in host larvae infected with the four *Leptopilina* wasps. The red and blue colors represent high to low expression levels based on the FPKM values.

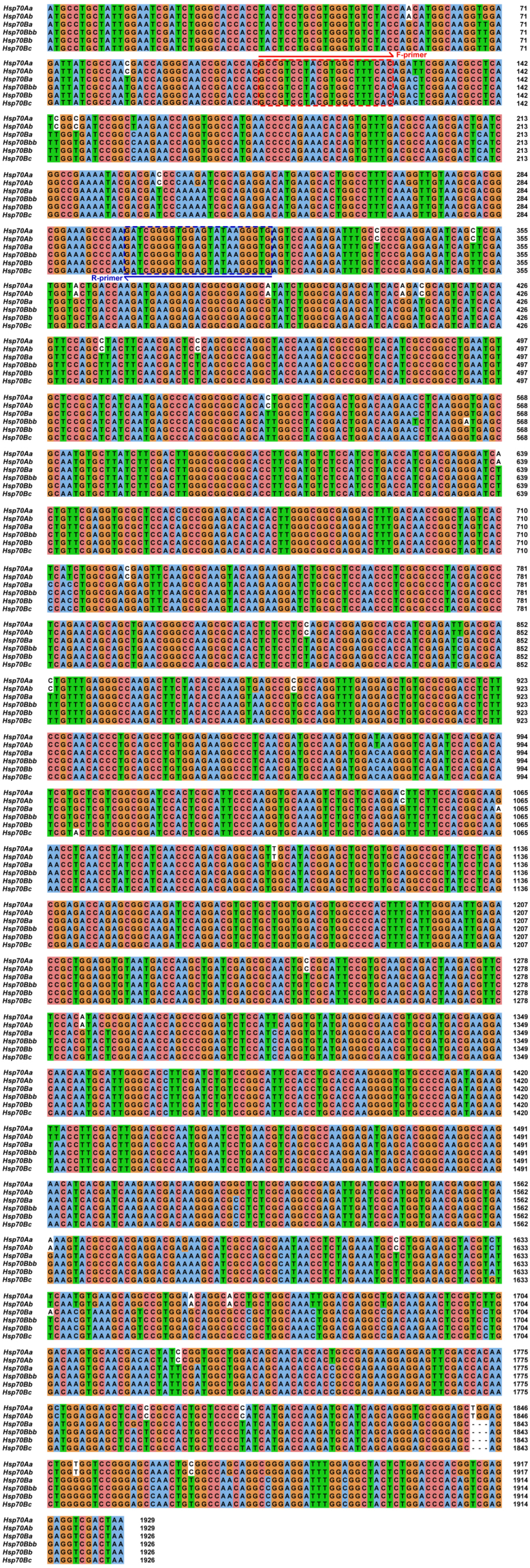

Fig. S7. Comparison of the coding sequences of *Hsp70* genes. Adenine (A) is highlighted in blue, thymine (T) is highlighted in green, guanine (G) is highlighted in orange, and cytosine (C) is highlighted in pink. The sequence in the red dashed box represents the forward primer, whereas the sequence in the blue dashed box represents the reverse primer for the qPCR analysis of *Hsp70* levels.

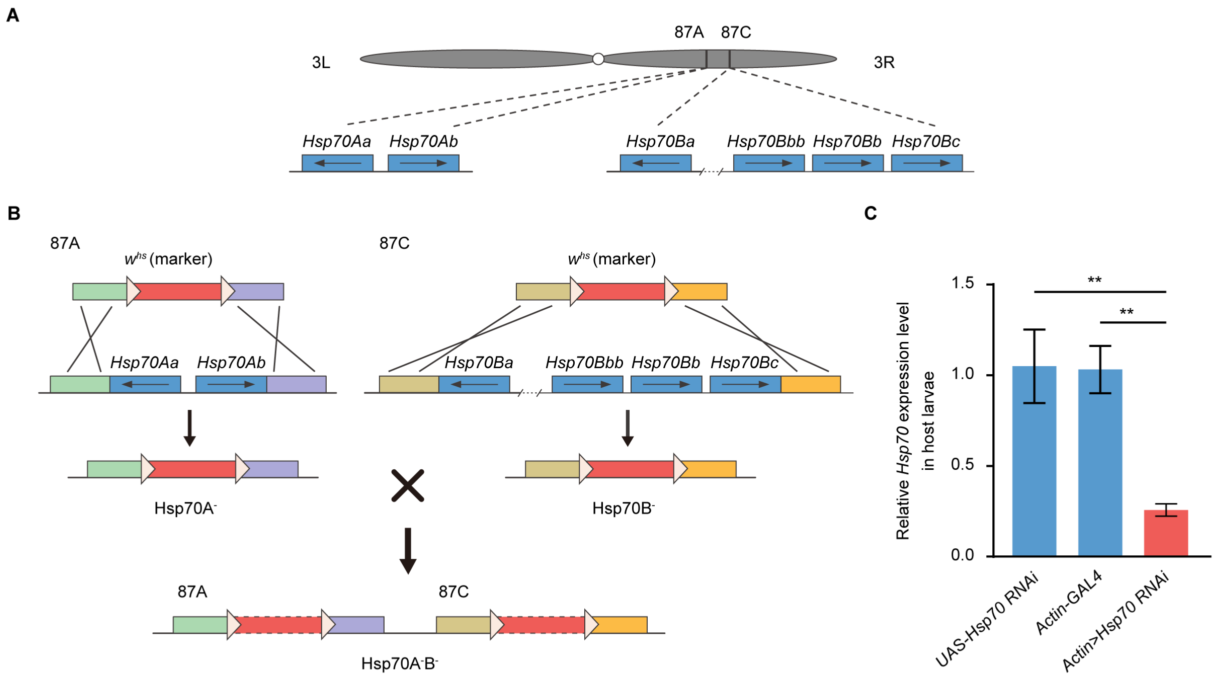

Fig. S8. *Hsp70* loss-of-function mutants and an *Hsp70* RNAi line. (A) Schematic diagram illustrating the organization of *Hsp70* genes in *D. melanogaster*. *Hsp70Aa* and *Hsp70Ab* are located at cytological locus 87A on the right arm of the 3rd chromosome, while *Hsp70Ba*, *Hsp70Bbb*, *Hsp70Bb* and *Hsp70Bc* are located at cytological locus 87C on the right arm of the same chromosome. Arrows represent transcriptional directions. **(B)** Schematic diagram illustrating the construction of *Hsp70* mutants, adapted from a previous study (*31*). Genomic deletions of *D. melanogaster Hsp70* genes were generated by homologous recombination. **(C)** Relative levels of *Hsp70* in different fly genotypes. The genotypes are *UAS-Hsp70 RNAi*, *Actin-GAL4*, *Actin>Hsp70 RNAi*. The experiments were performed with five biological replicates. The data are presented as the means ± SEMs. Significance was determined by one-way ANOVA with Tukey's multiple comparison test (**: $p < 0.01$).

A

	Hsp70Aa*	Hsp70Ab	Hsp70Ba*	Hsp70Bbb	Hsp70Bb	Hsp70Bc
Hsp70Aa	-	100%	98.60%	98.75%	98.91%	98.91%
Hsp70Ab	100%	-	98.60%	98.75%	98.91%	98.91%
Hsp70Ba	98.60%	98.60%	-	99.53%	99.69%	99.69%
Hsp70Bbb	98.75%	98.75%	99.53%	-	99.84%	99.84%
Hsp70Bb	98.91%	98.91%	99.69%	99.84%	-	100%
Hsp70Bc	98.91%	98.91%	99.69%	99.84%	100%	-

B

C

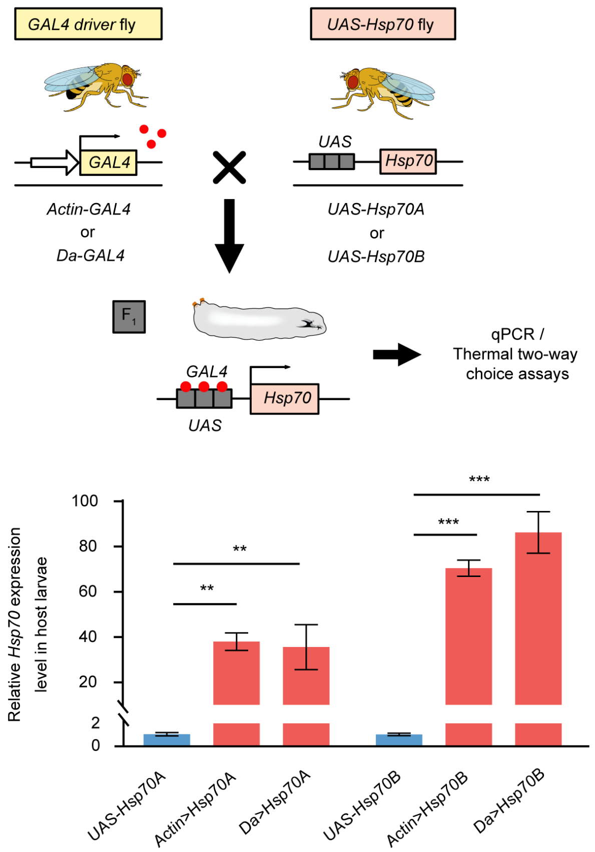

Fig. S9. Construction of transgenic *D. melanogaster* hosts overexpressing *Hsp70* genes. (A) Amino acid sequence alignment of *Hsp70* genes. Hsp70Aa was selected to generate *UAS-Hsp70A* transgenic flies, and Hsp70Bb was selected to generate *UAS-Hsp70B* transgenic flies. **(B)** Schematic diagram of *Hsp70A* or *Hsp70B* overexpression in *D. melanogaster* hosts via the *UAS-Hsp70A* and *UAS-Hsp70B* constructs in combination with *Actin-GAL4* and *Da-GAL4* drivers, respectively. **(C)** Relative levels of *Hsp70* in different fly genotypes. The genotypes are *UAS-Hsp70A*, *Actin>Hsp70A*, *Da>Hsp70A*, *UAS-Hsp70B*, *Actin>Hsp70B*, and *Da>Hsp70B*. The experiments were performed with five biological replicates. The data are presented as the means ± SEMs. Significance was determined by one-way ANOVA with Tukey's multiple comparison test (***: $p < 0.001$; **: $p < 0.01$).

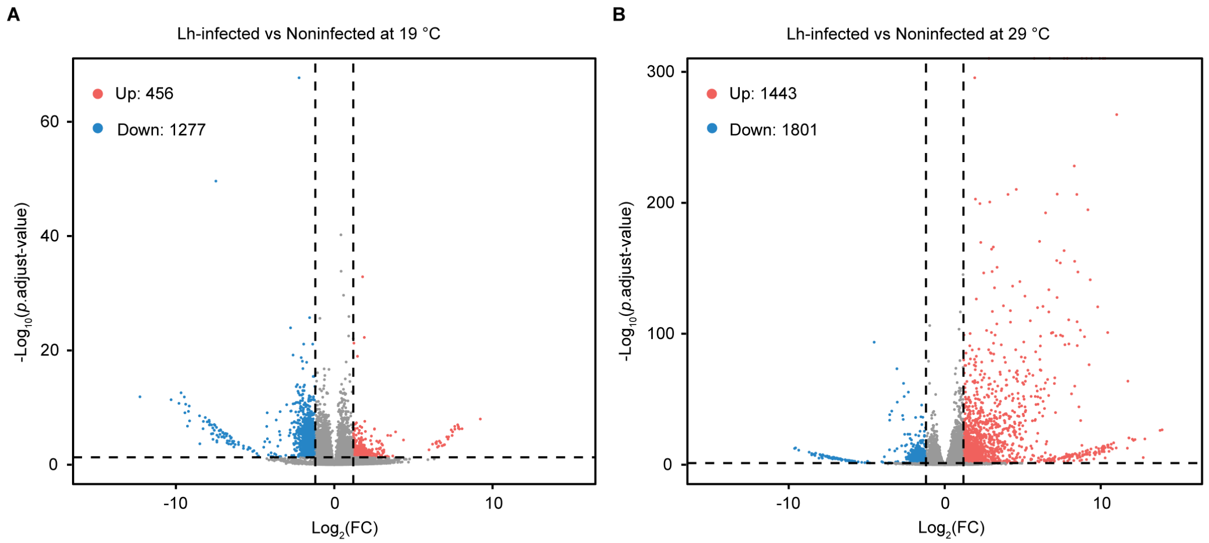

Fig. S10. Comparative transcriptomic analysis of Lh-infected and noninfected hosts. (A) Volcano plot of DEGs between the Lh-infected and noninfected samples at 19 °C. **(B)** Volcano plot of DEGs between the Lh-infected and noninfected samples at 29 °C. Noninfected: noninfected 1-day-old host pupae; Lh-infected: *L. heterotoma*-infected 1-day-old host pupae.

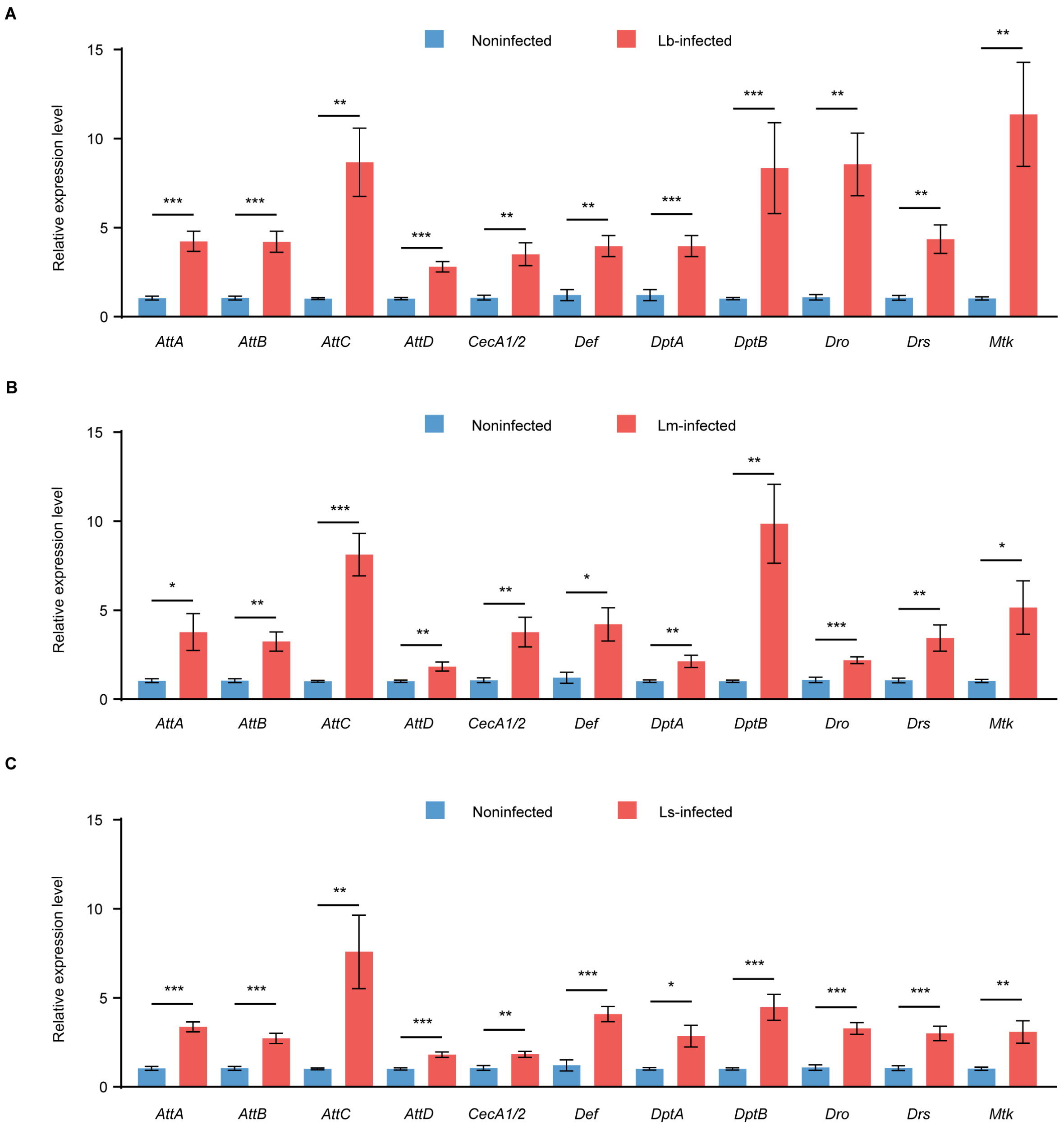

Fig. S11. Expression levels of *AMP* genes in fly hosts at 29 °C. (A-C) Relative mRNA levels of *AMP* genes in 1-day-old host pupae infected with *L. boulardi* (A), *L. myrica* (B) or *L. syphax* (C). Noninfected 1-day-old host pupae were used as controls. The experiments were performed with seven biological replicates. The data are presented as the means ± SEMs. Significance was determined by two-tailed unpaired Student's t test (***: $p < 0.001$; **: $p < 0.01$; *: $p < 0.05$).

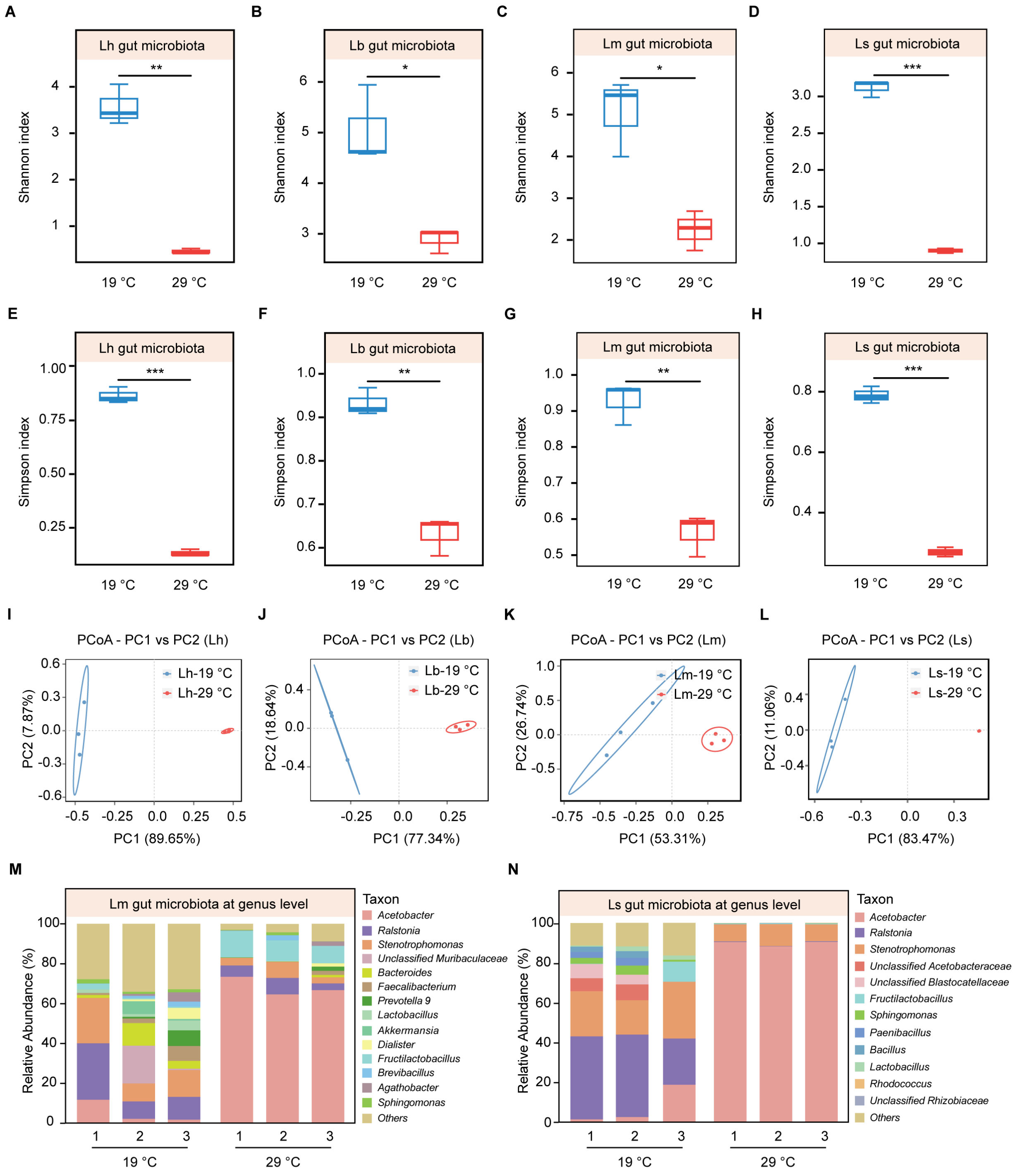

Fig. S12. Gut microbiota dysbiosis was detected in wasp larvae at high temperatures. (A-D) Shannon index of the gut microbiota in *L. heterotoma* larvae (A), *L. boulardi* larvae (B), *L. myrica* larvae (C) and *L. syphax* larvae (D) at 19 °C and 29 °C. The experiments were performed with three biological replicates. Significance was determined by two-tailed unpaired Student's t test (***: $p < 0.001$; *: $p < 0.05$). **(E-H)** Simpson index of the gut microbiota in *L. heterotoma* larvae (E), *L. boulardi* larvae (F), *L. myrica* larvae (G) and *L. syphax* larvae (H) at 19 °C and 29 °C. The experiments were performed with three biological replicates. Significance was determined by two-

tailed unpaired Student's t test (***: $p < 0.001$; **: $p < 0.01$). **(I-L)** Principal coordinate analysis (PCoA) of the gut microbiota in *L. heterotoma* larvae (I), *L. boulardi* larvae (J), *L. myrica* larvae (K), and *L. syphax* larvae (L) at 19 °C and 29 °C. **(M, N)** Bacterial community composition at the genus level of the gut microbiota in *L. myrica* larvae (M) and *L. syphax* larvae (N) at 19 °C and 29 °C.

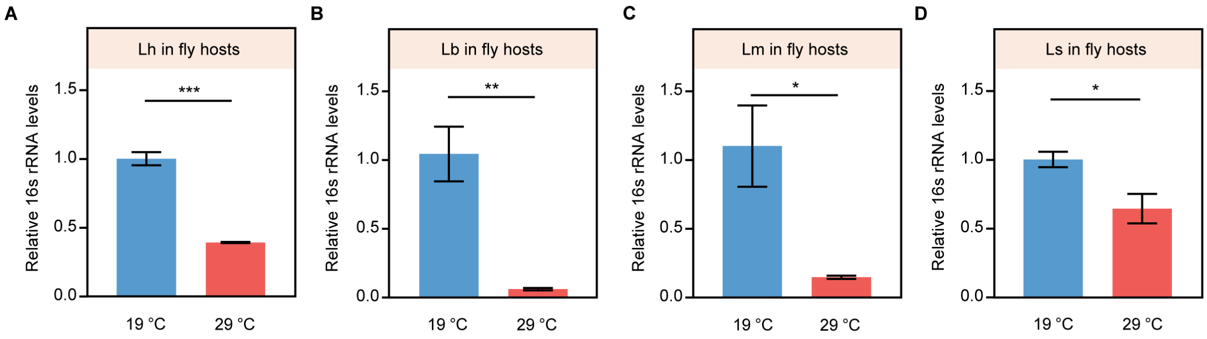

Fig. S13. The amount of bacteria in the gut of different *Leptopilina* wasps. **(A-D)** qPCR-based quantification of the number of gut bacteria in *L. heterotoma* larvae (A), *L. boulardi* larvae (B), *L. myrica* larvae (C), and *L. syphax* larvae (D) at 19 °C and 29 °C. The experiments were performed with three biological replicates. The data are presented as the means ± SEMs. Significance was determined by two-tailed unpaired Student's t test (***: $p < 0.001$; **: $p < 0.01$; *: $p < 0.05$).

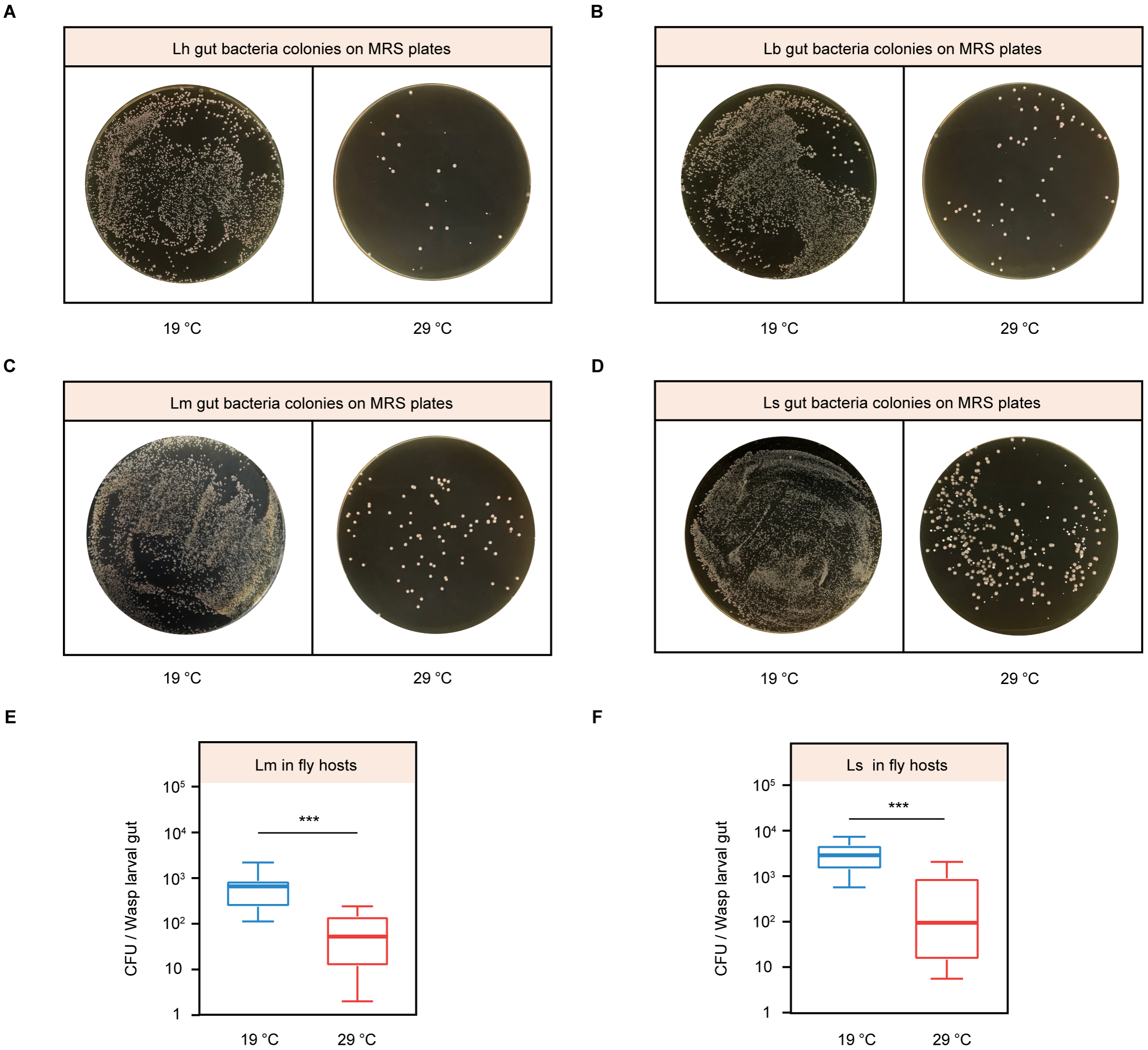

Fig. S14. CFUs of culturable gut bacteria on MRS plates. (A-D) Gut bacteria colonies on MRS plates from *L. heterotoma* larvae (A), *L. boulardi* larvae (B), *L. myrica* larvae (C) and *L. syphax* larvae (D) at 19 °C and 29 °C. **(E, F)** CFUs of gut bacteria in *L. myrica* larvae (E) and *L. syphax* larvae (F) at 19 °C and 29 °C. The experiments were performed with 26 biological replicates. Significance was determined by the Mann–Whitney U test (***: $p < 0.001$).

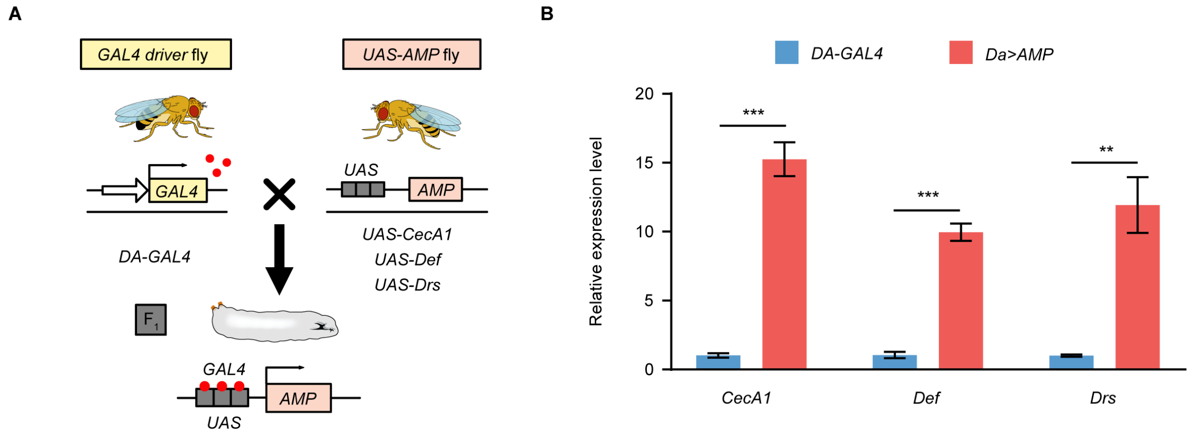

Fig. S15. Construction of *D. melanogaster* hosts overexpressing AMPs. (A) Schematic diagram of the overexpression of *AMPs* in *D. melanogaster* hosts via *UAS-CecA1*, *UAS-Def*, and *UAS-Drs* constructs in combination with the *Da-GAL4* driver. **(B)** Relative levels of *AMP* genes (e.g., *CecA1*, *Def* and *Drs*) in different genotypes. The genotypes are as follows: *Da-GAL4*, *Da>CecA1*, *Da>Def*, *Da>Drs*. The experiments were performed with three biological replicates. The data are presented as the means ± SEMs. Significance was determined by two-tailed unpaired Student's t test (***: $p < 0.001$; **: $p < 0.01$).

Supplementary Table legends

Table S1. The upregulated DEGs in transcriptome analysis of *Leptopilina*-infected host larvae vs Noninfected host larvae.

Table S2. The downregulated DEGs in transcriptome analysis of *Leptopilina*-infected host larvae vs Noninfected host larvae.

Table S3. GO enrichment analysis of upregulated DEGs in comparison of transcriptome data for Lh-infected host pupae and Noninfected host pupae at 29 °C.

Table S4. GO enrichment analysis of upregulated DEGs in comparison of transcriptome data for Lh-infected host pupae and Noninfected host pupae at 19 °C.

Table S5. The upregulated immune-related genes in Lh-infected host pupae at 29 °C.

Table S6. The increased Hsp70 expression in infected ectotherm animals.

Table S7. List of primers used in this study.
